# Clinical and molecular characteristics of carbapenem non-susceptible *Escherichia coli*: A nationwide survey from Oman

**DOI:** 10.1371/journal.pone.0239924

**Published:** 2020-10-09

**Authors:** Hissa M. Al-Farsi, Angela Camporeale, Karolina Ininbergs, Saleh Al-Azri, Zakariya Al-Muharrmi, Amina Al-Jardani, Christian G. Giske

**Affiliations:** 1 Division of Clinical Microbiology, Department of Laboratory Medicine, Karolinska Institutet, Stockholm, Sweden; 2 Central Public Health Laboratories, Ministry of Health, Muscat, Oman; 3 Department of Clinical Microbiology, Karolinska University Hospital, Stockholm, Sweden; 4 Department of Clinical Microbiology, Sultan Qaboos University Hospital, Muscat, Oman; Zhejiang University, CHINA

## Abstract

The prevalence of carbapenem-resistant *Enterobacterales* (CRE) in the Arabian Peninsula is predicted to be high, as suggested from published case reports. Of particular concern, is carbapenem-resistant *E*. *coli* (CR-EC), due to the importance of this species as a community pathogen. Herein, we conducted a comprehensive molecular characterization of putative CR-EC strains from Oman. We aim to establish a baseline for future molecular monitoring. We performed whole-genome sequencing (WGS) for 35 putative CR-EC. Isolates were obtained from patients at multiple centers in 2015. Genetic relatedness was investigated using several typing approaches such as MLST, SNP calling, phylogroup and CRISPR typing. Maxiuium likelihood SNP-tree was performed by RAxML after variant calling and removal of recombination regions with Snippy and Gubbins, respectively. Resistance genes, plasmid replicon types, virulence genes, and prophage were also characterised. The online databases CGE, CRISPRcasFinder, Phaster and EnteroBase were used for the *in silico* analyses. Screening for mutations in genes regulating the expression of porins and efflux pump as well as mutations lead to fluoroquinolones resistance were performed with CLC Genomics Workbench. The genetic diversity suggests a polyclonal population structure with 21 sequence types (ST), of which ST38 being the most prevalent (11%). SNPs analysis revealed possible transmission episodes. Whereas, CRISPR typing helped to spot outlier strains belonged to phylogroups other than B2 which was CRISPR-free. The virulent phylogroups B2 and D were detected in 4 and 9 isolates, respectively. In some strains bacteriophages acted as vectors for virulence genes. Regarding resistance to β-lactam, 22 were carbapenemase producers, 3 carbapenem non-susceptible but carbapenemase-negative, 9 resistant to expanded-spectrum cephalosporins, and one isolate with susceptibility to cephalosporins and carbapenems. Thirteen out of the 22 (59%) carbapenemase-producing isolates were NDM and 7 (23%) were OXA-48-like which mirrors the situation in Indian subcontinent. Two isolates co-produced NDM and OXA-48-like enzymes. In total, 80% (28/35) were CTX-M-15 producers and 23% (8/35) featured AmpC. The high-risk subclones ST131-H30Rx/C2, ST410-H24RxC and ST1193-H64RxC were detected, the latter associated with NDM. To our knowledge, this is the first report of ST1193-H64Rx subclone with NDM. In conclusion, strains showed polyclonal population structure with OXA-48 and NDM as the only carbapenemases in CR-EC from Oman. We detected the high-risk subclone ST131-H30Rx/C2, ST410-H24RxC and ST1193-H64RxC. The latter was reported with carbapenemase gene for the first time here.

## Introduction

Infections caused by *E*. *coli* comprise a huge public health problem [1]. Particularly this is true for infections caused by carbapenem-resistant *E*. *coli* (CR-EC), as carbapenems are among the last-resort treatment options for multidrug-resistant strains [2]. Carbapenem resistance can occur due to an interplay between a number of mechanisms including intrinsic mutations in existing genes, leading to either overexpression of efflux pumps or downregulation of porins. Another mechanism is the acquisition of novel genes encoding carbapenem-hydrolysing enzymes such as *bla*_KPC_, *bla*_NDM_ and *bla*_OXA-48-like_ by horizontal gene transfer (HGT), largely due to plasmids [3]. Phages also play an important role in the HGT. However, their contribution to the spread of antibiotic resistance genes (ARGs) remains inconclusive, with conflicting evidence in the literature [4–8].

Counteracting HGT, the acquisition of plasmids and phages in bacteria is restricted by Clustered Regularly Interspaced Short Palindromic Repeats (CRISPR) [9]. CRISPR and CRISPR-associated genes (CRISPR-cas) system consists of *cas* genes, direct repeats (DR) and spacers [10]. Spacers contain part of the invaders DNA sequences to immunize the bacteria against them [11]. Additionally, CRISPR has been suggested as a typing tool in which related strains share similar spacers [12, 13]. In *E*. *coli*, CRISPR-cas system consists of I-E and I-F subtypes. I-E subtype is the commonest with two main loci—CRISPR 2.1 and CRISPR 2.3, as well as occasionally CRISPR 2.2 locus. While, the I-F subtype is less frequent with two loci—CRISPR 4.1 and CRISPR 4.2 [14, 15].

Multilocus sequence typing (MLST) of resistant strains is an important approach to study bacterial clonality and hence to trace an outbreak. Also, it helps to recognise particular high-risk clones (HiRC) that originate from single bacterial strains yet have the potential for global expansion [16]. In *E*. *coli*, the commonest HiRC is ST131-H30Rx/C2. It is fluoroquinolone-resistant and contributed to the dissemination of CTX-M-15 globally [17]. Worryingly, it was reported with carbapenemase in the UK recently [18]. Another emerging HiRCs is ST1193-H64Rx. It is resistant to fluoroquinolones and to multiple antibiotics including cephalosporins [19]. Both subclones belong to the virulent B2 phylogroup. A convergence of virulent HiRC with antibiotic resistance determinants, particularly carbapenemase genes, is of great concern to public health globally due to their ability to spread rapidly.

In Oman, infection caused by CRE are notifiable under the communicable disease act. For reporting purposes, CRE are defined as either resistant to meropenem (MIC ≥4 mg/L) or ertapenem (MIC ≥2 mg/L), based on susceptibility testing at the reporting facility or by the production of a carbapenemase demonstrated using a recognised test according to Clinical and Laboratory Standards Institute (CLSI) guidelines [20, 21]. Based on this definition, the incidence of CRE infection in 2018 was 11 per 1,000 bacteremic patients, compared to 12 the year before. However, the mortality rate almost doubled from 32% to 62% [22]. A report from the World Health Organization (WHO)’s Global Antimicrobial Resistance Surveillance System (GLASS) stated that 2% of 261 tested *E*. *coli* from Oman were CR-EC in 2017 [23]. Although a few reports are available on the antibiotic resistance profiles of CR-EC in Oman, there is a paucity of studies on other genetic characteristics such as the plasmid and virulence profiles as well as clonality.

In this study, we sought to unravel the genetic background of CR-EC from Oman with several *in silico* typing approaches such as SNPs, MLST, phylogroup, serotypes and CRISPR typing. Moreover, we studied their resistome, plasmidome, virulome, and prophage contents. Lastly, we investigated possible transmission episodes, by high-resolution epidemiological typing data.

## Materials and methods

### Bacterial isolates and phenotypic characterisation

As a part of the National Health Policy in Oman, clinical isolates with reduced susceptibility (I or R) to carbapenems, are identified as putative CR-EC and shall be sent to the Public Health Laboratories for further analyses. Herein, we studied 35 *E*. *coli* isolates which were initially reported with reduced susceptibility to carbapenem according to CLSI guidelines [21]. They were collected from January to October 2015. Demographic data were obtained from clinical data repositories complementing the strain collection forms.

Strains were further investigated at Karolinska Institutet for a wide range of antibiotics with disk diffusion (imipenem, meropenem, ertapenem, cefotaxime, ceftazidime, aztreonam, piperacillin-tazobactam, ciprofloxacin, amikacin, gentamicin, ceftazidime-avibactam), agar dilution (fosfomycin) and broth microdilution (colistin, tigecycline) according to EUCAST guidelines [24]. Diagnostic kits from ROSCO Diagnostica A/S (Taastrup, Denmark) were used to classify carbapenemases into MBL, OXA-48-like or ESBL. The kits, which is based on the combination disk testing principle, consist of cartridges with ready-to-use tablets that classify carbapenemases based on their response to chemical inhibitors phenotypically. Rapidec Carba NP (bioMérieux SA, Marcy-l'Étoile, France) was used to test the production of carbapenemase enzymes as described previously [25].

### Whole genome sequencing

Genomic DNA was extracted with the MagNA Pure 96 system (Roche Applied Science, Manheim, Germany) automated DNA-extraction system, following the manufacturer’s guidelines. The quantity of the extracted DNA was measured using Qubit dsDNA assay kit (Life Technologies Europe, Bleiswijk, The Netherlands). Next, WGS was performed with Illumina Nextera Library Prep kits using HiSeq 2500 (Illumina, San Diego, USA) at the Science for Life Laboratory (SciLifeLab, Solna, Sweden) generating 2×100 paired-end sequences. Sequencing quality control showed that 30x coverage was >80% for 74% of isolates. Mean duplication rate was 4.7% (SD 2.5) and mean median insert size was 327 (SD 201) (S1 Table). Raw reads data generated in this study were deposited in SRA as project PRJNA544438 (accession no. SAMN11840190-SAMN11840224).

The generated raw reads were uploaded to the EnteroBase web resource. Triming, *de novo* assembly, quality control and annotation were performed through the associated EnteroBase pipeline [26]. Mean N50 and N90 of the assembled genomes were 151,472 (SD 42,739) and 31,424 (SD 8,405), respectively. The mean length of genome size was 5,027,470 (SD 174,295) while the mean of contigs number was 162 (SD 62) (S1 Table).

Furthermore, the downloaded draft assemblies were remapped to short reads back onto the assembly using the BWA-MEM algorithm of BWA v0.7.17. Next, the output SAM files were sorted to BAM files using SAMtools v0.1.9 and finally polished with Pilon v1.23 to fill gaps and fix local misassemblies [27–29]. In the improved assemblies, a total reduction of 29% and 26% were observed in number of gaps and N-counts, respectively. Five strains were gap- and N-free (S1 Table). The genomic quality data were either obtained from EnteroBase web resource or calculated by assembly-stats v1.0.1 tool [30].

### Strains typing and SNPs calling

We used different typing approaches to reveal the genetic background of the studied strains. For MLST typing, EnteroBase web resource was used to obtain classical MLST (MLST), core-genome MLST (cgMLST) and whole-genome MLST (wgMLST) [31]. Also, **we used the same database** to obtain O:H serotypes. While for phylogroup, the assembled genomes were uploaded to the ClermonTyping v1.4.0 web tool as described previously [31, 32].

Furthermore, variant calls for SNP analysis were performed using Snippy v4.4.5 against the chromosome of *E*. *coli* O55:H7 str. RM12579 (CP003109.1) as it was the closest common ancestor to the studied strains [33]. Alignment file was filtered from variants with elevated densities of base substitutions as a putative recombination events by Gubbins v2.4.1 with a threshold to exclude taxa with >30% missing data [34]. Since the reference strain belonged to a different ST type (ST335), we masked the seven house-keeping genes used for MLST typing, to avoid false correction by Snippy and false-positive recombination sites by Gubbins yet they were unmasked after filtering out recombination sites. Then SNP-sites v2.5.1 was used to reduce the filtered alignment to the core polymorphic sites [35]. Next, the core alignment output was used to create SNP-matrix by snp-dists v0.7.0 (https://github.com/tseemann/snp-dists) as well as to create randomised accelerated maximum likelihood (RAxML) tree by RAxMLv8.2.12 under GTR-GAMMA model bootstrapping (1,000 replicates) with vectorised AVX2 instructions [36]. The tree layout was graphically edited using iTOL v5.6 [37]. Allelic difference with ≤ 10 SNPs to the nearest neighbour was used as a cut-off value for clonal relatedness [38].

Additionally, CRISPR loci and *cas* genes identification were performed using CRISPRcasFinder server (accessed on 16^th^ July 2020) while *cas* gene detection was performed via subtyping clustering model option [39, 40]. CRISPRcasFinder gives evidence level for each CRISPR locus as 1 being less evident. We reported only CRISPR with evidence level ≥ 4 or those with <4 but harbouring *cas* genes. CRISPR loci were defined based on flanking genes as CRISPR 2.1 flanked by *cys*H and *iap*, CRISPR 2.3 and CRISPR 2.2 sandwiched between *ygc*F and *ygc*E, while CRISPR 4.1 just after *clp*A and CRISPR 4.2 just before *inf*A. Annotated genomes obtained from EnteroBase were used to allocate the arrays for the classification and for direction verification. To screen for anti-CRISPR and self-target spacer, we used CRISPRminer database [41]. Also, spacers were aligned against viruses and plasmids using BLASTn to determine similarity with phages and plasmids. An Expect value (E-value) of 0.0001 or less was used to identify matches. The spacers were considered similar, if their sequences were at least 90% identical.

### Analysis of accessory genes

Assembled genomes were uploaded to the Center for Genomic Epidemiology (CGE) database. Resistance genes were screened with a threshold of 100% identity over 100% coverage using ResFinder v1.3 web tool [42]. We looked particularly for ARGs against β-lactam, yet ARGs against other broad spectrum of antibiotic classes (aminoglycosides, fluoroquinolones, tetracycline, folate synthesis inhibitors and phenicol) were also reported. Additionally, reads were aligned against K-12 (NC_000913.3) to identify missense mutations in genes related to fluoroquinolone resistance (*par*E, *par*C and *gyr*A) as well as in genes regulating AcrAB-TolC efflux pump (*acr*R, *mar*A, *mar*B, *mar*R, *sox*S, *sox*R, *env*Z) and porins (*omp*C, *omp*F, *omp*R) using CLC Genomics Workbench 20.0 (Qiagen Bioinformatics, Aarhus, Denmark).

Furthermore, to reflect on plasmidome contents, we used *in silico* plasmid replicon (rep) typing approach as described previously [43]. We uploaded the assembled genomes to PlasmidFinder v1.3 web tool with default setting (95% identity and 60% minimum length) and subtyped frequent replicons (IncF, IncI1, IncN) with plasmid MLST using pMLST v1.4 web tool as described previously [44]. To identify mobile genetic elements (MGEs) and their relation to carbapenemase genes we uploaded the assembled genomes into MobileElementFinder v1.0.2 within CGE database with default setting (95% minimum alignment coverage, 90% identity and 30 nt maximum truncation).

Additionally, virulence genes and *fim*H type from assembled genomes were performed with default settings (90% identity and 60% minimum length) using VirulenceFinder 2.0 and FimTyper 1.0 (95% identity) web tools, respectively, as described previously [45].

Finally, prophage typing was performed using the Phaster database (accessed on 18 July 2020) [46, 47]. Phaster categorises prophages based on their score into three groups; intact (score > 90), incomplete (score 90–70) and questionable (score <70). The obtained sequences were screened for ARGs and virulence genes using CGE web tools as described previously [48].

### Ethical statement

Ethical approval was obtained from Ministry of Health in Oman to utilise the described bacterial isolates and collected (anonymised) patients’ data for this study (MH/DG/R&S/32/2015).

## Results

### Demographic data

The samples were obtained from Oman, which is a country located in the Arabian Peninsula. A total of 35 positive isolates in the initial screening for CR-EC were collected from eight tertiary care hospitals in Oman between January and October 2015 (S1 Fig). The majority of them (n = 20/35) were from Khoula Hospital (S2 Table). It is a tertiary care unit specialised in bone injury including car accidents which are a serious public health problem in Oman [49].

The majority of isolates were clinical (n = 22/35) with wound being the predominant source (Table 1). The remaining isolates were from screening (n = 13/35). The screening isolates were obtained as per infection prevention and control guidelines in Oman. This includes all cases admitted previously in hospitals within Oman or abroad for the last three months or 6 months, respectively. Also, all cases admitted in intensive care unit (ICU) or those exposed to positive CRE cases are screened as per the guidelines. The source of screening isolates varied from rectal and wound swabs to catheter sites and tracheal secretions if patients were intubated.

**Table 1:** Summary of clinical and genetic data for the 35 *E*. *coli* isolates from Oman.

**Demographics**	
**Source**	
Hospital	Khoula (57%), Royal (14%), SQH (11%), SQUH (3%), Sohar (6%), Buraimi (3%), Nahda (3%), Sur (3%)
Nationality	Omani (68.6%), Yemeni (25.7%), Indian (5.7%)
Purpose	screening (37%), clinical (63%)
Specimen	fecal screening (37%), wound (26%), urine (14%), blood (6%), biopsy (9%), respiratory (6%)
**Patient information**	
Age (years)	mean (44), median (55), range (3 weeks-81 years)
Sex	male (71%), female (29%)
Survival	86%
Antibiotic usage	meropenem (29%), β-lactam inhibitor (43%), 3rd generation cephalosporin (17%), penicillin (11%)
	fluoroquinolones (23%), gentamicin (9%), colistin (11%)
Travel information	India (29%)
Bacterial phylogenetics and sequence types (n*)	
Group A (12)	ST1702 (2), ST167 (3), ST617 (3), ST46 (1), ST540 (1), ST361 (2)
Group B1 (4)	ST101 (2), ST448 (1), ST156 (1)
Group B2 (4)	ST131 (1), ST73 (1), ST127 (1), ST1193 (1)
Group C (4)	ST410 (3), ST652 (1)
Group D (9)	ST38 (4), ST405 (3), ST2914 (1), ST2659 (1)
Group F (2)	ST1340 (1), ST6870 (1)
CRISPR-cas system (m**)	
I-E subtype (m)	CRISPR 2.3 (30), CRISPR 2.1 (29)
I-F subtype (m)	CRISPR 4.1 (5), CRISPR 4.2 (1)
Direct repeat	4 groups
Spacers (Total = 637)	unique (144), self-targeting (1), matching phage (28), matching plasmid (10)
*cas* genes (m)	I-E subtype: present fully (25) present partially (4) absent (1)
	I-F subtype: present fully (1), absent (4)
**β-lactamase genes and plasmid replicons (m)**	
**β-lactamase genes (m)**	
Carbapenemases (24)	NDM-5 (8), NDM-1 (5), NDM-4 (1), NDM-7 (1), OXA-181 (5), OXA-48 (4)
ESBLs (32)	CTX-M-15 (28), CTX-M-24 (1), CTX-M-3 (1), CTX-M-55 (1), SHV-12 (1)
AmpC (13)	CMY-42 (7), CMY-2 (3), CMY-6 (1), DHA-1 (2)
Penicillinase (44)	TEM-1B (24), OXA-1 (18), OXA-9 (2)
**Plasmid Replicons (Total = 136)**	
Incompatibility groups (116)	IncFIA (23), IncFII (30), IncFIB (23), IncX (14), IncI1 (11), IncA/C2 (1), IncL/M (3), IncN (3), IncY (2),
	IncQ1 (5), IncR (1)
Colicin (19)	BS512 (9), ColKP3 (3), ColpVC (4), Col156 (3)
Others (1)	p0111 (1)
**Virulence genes and prophages (m)**	
**Virulence genes (Total = 380)**	
Adhesion (73)	*fim*H (24), *air* (10), *eil*A (10), *iha* (3), *lpf*A (10), *pap* (5), *foc* (1), *afa* (3), *yfc*V (6), *sfa (*1)
Toxins (32)	*ast*A (2), *cnf*1 (2), *clb*B (2), microcin genes (7), colicin genes (5), *sen*B (6), *pic* (2), *sat* (3), *vat* (3)
Siderophores (95)	*iro*N (2), *iuc*C (12), *iut*A (12), *irp*2 (17), *fyu*A (17), *chu*A (15), *sit*A (20)
Protectin (162)	*kps*E (15), *kps*MII (10), *neu*C (3), *gad* (34), *usp* (4), *iss* (21), *ter*C (34), *tra*T (23), *hra* (15), *tcp*C (3)
Others (18)	*cap*U (11), *omp*T (7)
**Prophages (Total = 200)**	
Detection (%)	intact (40%), questionable (12%), incomplete (48%)
Virulence genes (m)	*iss* (14), *irp*2 (1), *kps*E (1), *kps*MII (1)

*n: total number of geneome with the respective attribute.

**m: total number of attribute presented in the whole genomic collection.

The majority of the strains were isolated from male patients (n = 25/35). Median age of the patients was 50 years (IQR = 20–66). Five patients expired, of whom two had confirmed bacteraemia (S2 Table). The data regarding antibiotic usag by the source patients were available for 32 samples. Nine of them were treated with meropenem and seven were treated with extended-spectrum cephalosporins. Ten patients (29%) had a travel history to India.

### Genetic diversity showed a polyclonal population structure

**Fig 1:**
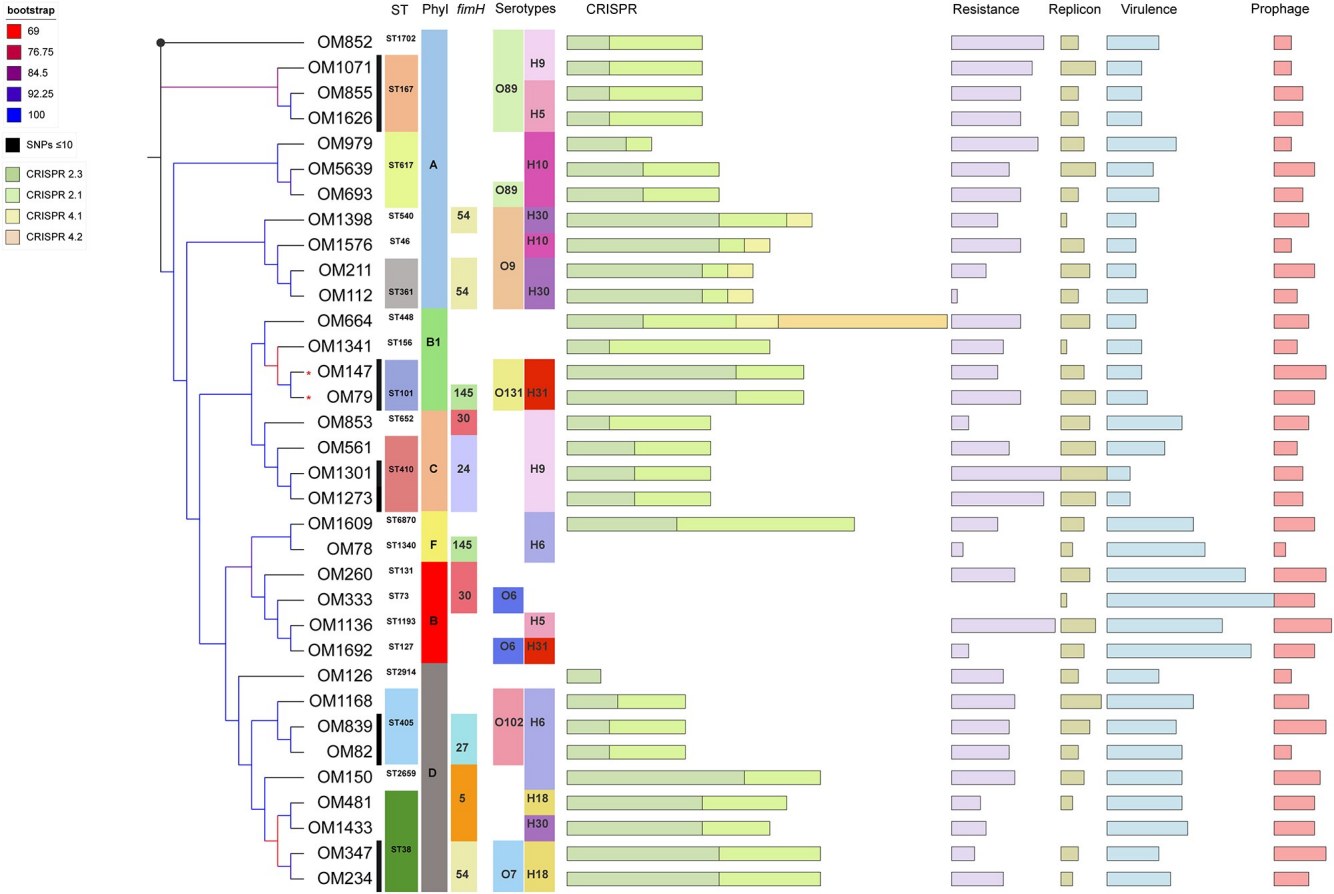
Genetic diversity for the studied strains. The maximum likelihood tree was inferred with 1,000 bootstrap based on 25,534 core SNPs. Sample ID is indicated in the tip of each branch. Since OM852 and OM898 strains were identical in SNPs analysis, only one strain (OM852) is presented here; marked with black dot at the branch. Serotypes and *fim*H variants were presented only if occured more than once. Bars represent total number of spacers in 4 CRISPR arrays, resistance genes in 6 antibiotic classes (β-lactams, aminoglycosides, fluoroquinolones, tetracyclines, folate synthesis inhibitors and phenicols), plasmid replicons, virulence genes and prophages. Asterisk indicates strains obtained from the same patient.

To unravel population structure and genetic diversity, different typing approaches were used including SNPs, MLST, phylogenetic and CRISPR typing. Core SNPs analysis was inferred based on 25,534 input positions remained after filtering (Fig 1). The core SNPs diffrences ranged between 0 (OM852 and OM898) to 8,031 (OM82 and OM260). Clonality between individual strains (SNP≤10) was observed on several occasions (Fig 1).

Regarding MLST, both cgMLST and wgMLST resulted in unique STs for each strain, which did not help to recognise genetic relatedness between the studied strains. Classical MLST, based on 7 house-keeping genes, revealed 22 sequence types (STs) with ST38 being the most common as it was detected in 4 isolates (Table 1). Other STs were ST167, ST617, ST405 and ST410 which were detected in 3 strains each. The majority of STs were singletons. This reflects a polyclonal popluation structure with wide genetic diversity as expected since the samples were obtained for surveillance purpose rather than outbreak investigation.

Furthermore, 6 phylogenetic groups were detected. The majority of isolates belonged to phylogroup A (12/35). The virulent B2 group was detected in four isolates. Also, D and F phylogroups were detected in 9 and 2 isolates, respectively (Table 1 and Fig 1).

**Fig 2:**
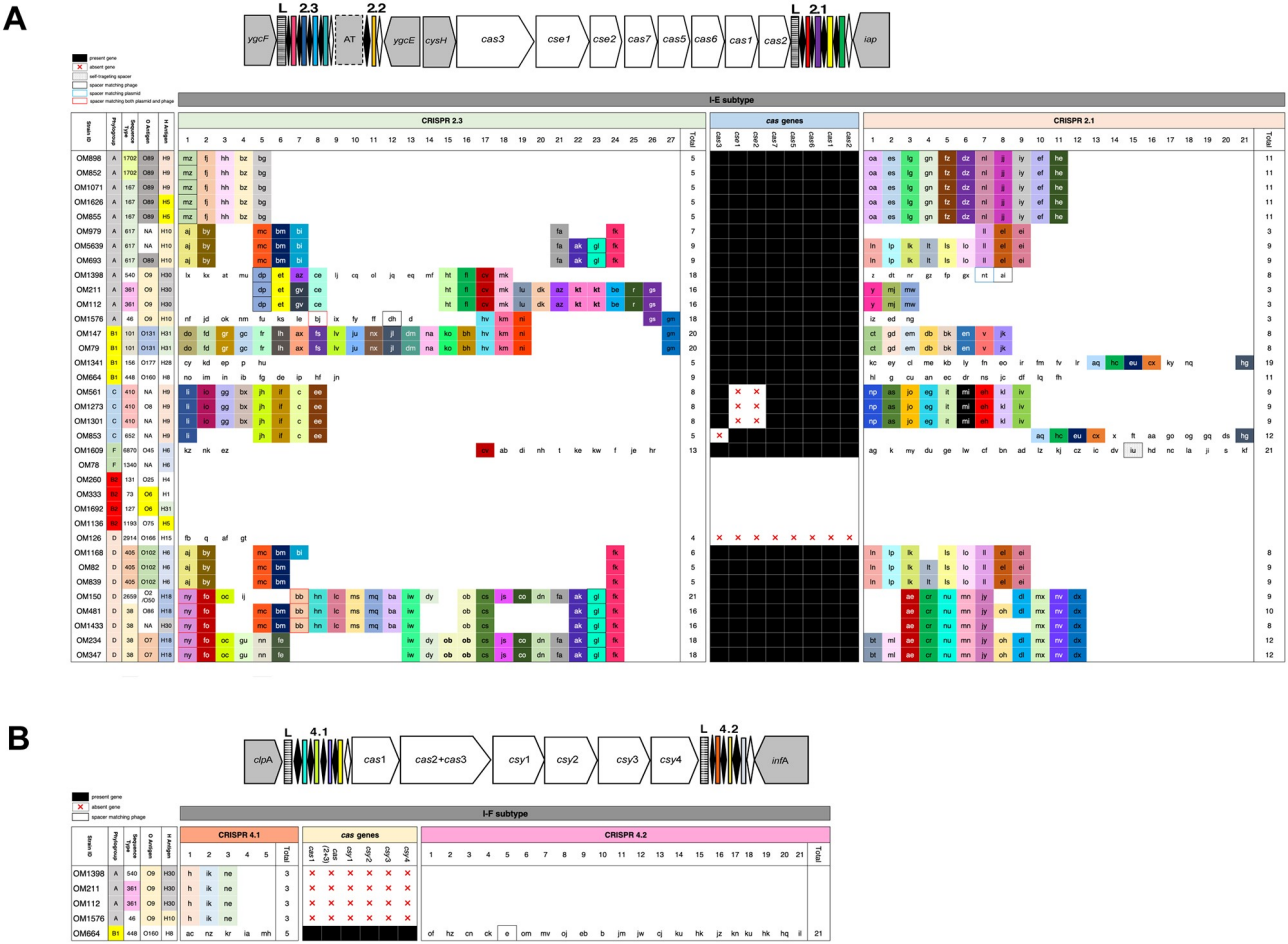
CRISPR-cas system for the 35 *E*. *coli* strains. Graphic representation of CRISPR-cas system of I-E (A) and I-F (B) subtypes. In the structural demonstration gray boxes represented genes not part of CRISPR-cas system. Boxes point towards direction of transcription. Within CRISPR locus, leader represented by “L”. Direct repeats depicted with black diamond and spacers with coloured rectangles. Strains were ordered based on SNPs analysis while spacers were ordered as per their occurance in a positive strand direction in each strain. Identical colours and letters present identical spacers while unique spacers have white background. Letters in bold font represent spacers occurred more than once in the same loci.

CRISPR typing differentiates strains within the same phylogroup into subclusters. For example, within phylogroup A we saw three different clusters (Fig 2). Yet, MLST had higher resolution than CRISPR typing. For example, ST1702 and ST167 both belong to clonal complex 10 and had identical CRISPR-cas system (Figs 1 and 2). Interestingly, all four strains with B2 phylogroup lacked CRISPR as well as one strain of F phylogroup, known to be genetically close to B2 phylogroup.

We identified 65 CRISPR loci in 30 isolates (Table 1). Within I-E subtype, 29 strains had both 2.3 and 2.1 loci while one harboured CRISPR 2.3 only (OM126). Yet, CRISPR 2.2 locus could not be detected in any strain. Regarding I-F subtype, five strains only had this subtype with one isolate having both 4.1 and 4.2 loci (OM664).

The presence of CRISPR system does not always indicate an active system. To reflects on its activity, we studied two factors. First, the presence of *cas* genes as this most likely suggests an active CRISPR-cas system (Fig 2). Within strains having I-E subtype, we found 25 complete, 4 partial, and one isolate with no trace of *cas* genes. The latter had one array only (CRISPR 2.3) with 4 spacers which might suggest decaying system. Regarding I-F subtype, 4 strains had no *cas* geneswhile they still harboured *cas* genes at I-E subtype (Fig 2). Interestingly, OM664 had a complete set of *cas* genes in both I-E and I-F subtypes, reflecting an active CRISPR-cas system.

The second factor that reflects the activity of CRISPR-cas system is the number of spacers. Here, 637 spacers were detected, among which 144 (23%) were unique (Fig 2). The highest number of spacers (n = 46) was observed in OM644 (ST448 and phylogroup B1). All of them were unique spacers whereas the majority of strains shared at least one spacer with each other. This indicates a distinct genetic background for this particular strain (OM664). Interestingly, it was isolated from a patient with a recent travel history to India (S2 Table). Hence, CRISPR particularly in *E*. *coli* might be a useful tool to recognise outlier strains with distinct genetic background. Unexpectedly, identical spacers occurred more than once in the same CRISPR loci within the same strain. For example, OM211 and OM347 both had two copies of a particular spacer named kt and ob, respectively (Fig 2). This is uncommonly seen in CRISPR system and we could not exclude this might be due to technical error related to sequencing or assembly. Additionally, we investigated if spacers had genetic interaction with phages and plasmids. We found 28 spacers matching phages and 10 matching plasmids while one spacer was matching bacterial chromosome as self-targeting (Fig 2). Worth noting, strains with identical spacers, tend to have the same serotype (Fig 2). For example three out of four strains with identical spacers at I-F subtypes, were O9:H30 (Fig 2). Similar finding was reported previously [15].

For serotyping, we used an *in silico* approach. While H-antigens could be identified in all strains, O-antigens could not be predicted in 7 strains. We could identify 12 H-types with H9 being the commonest (n = 7), followed by H6 (n = 5). On the other hand, 15 O-types could be seen with O89 being the most common (n = 6), followed by O9 (n = 4). O- and H-antigens revealed 19 different serotypes, with O89:H9, O9:H30 and O102:H6 being slightly predominant in 3 isolates each (Figs 1 and 2).

### Mechanisms of carbapenem resistance

**Fig 3:**
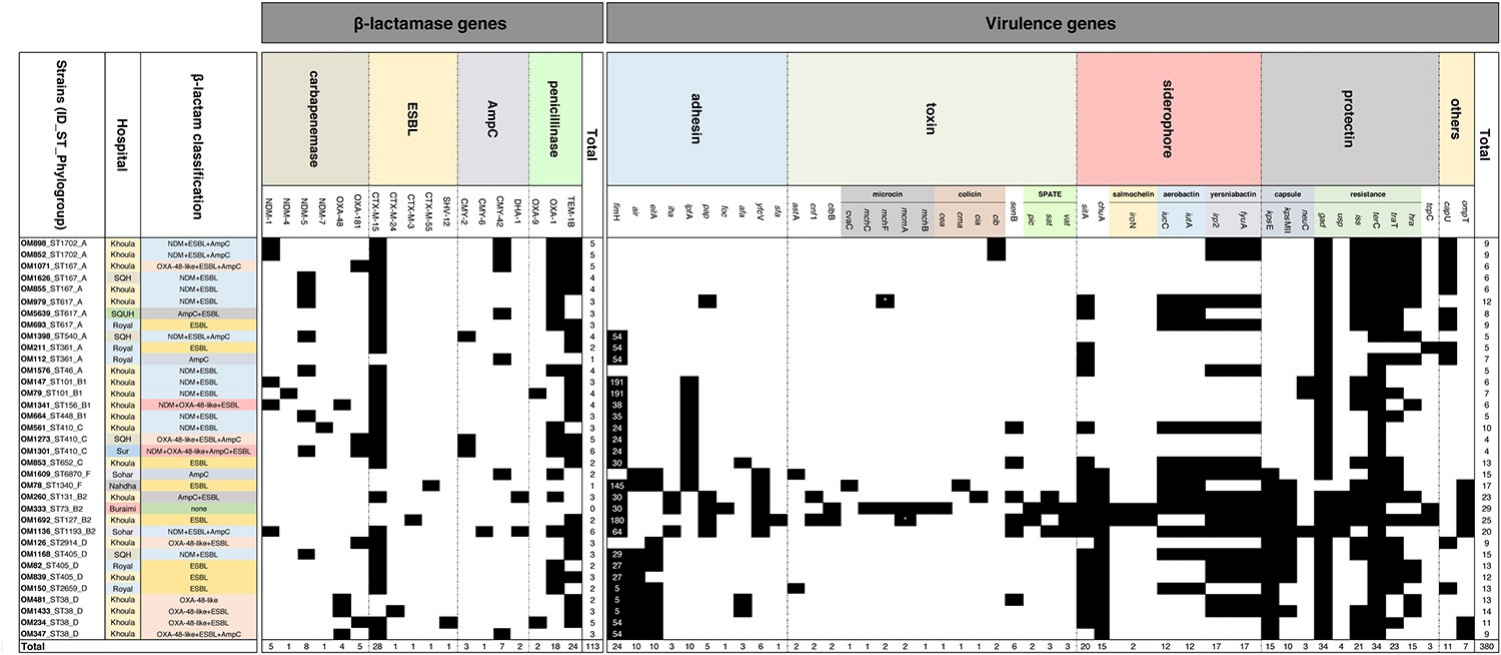
Heatmap presents *in silico* analyses of β-lactamase and virulence genes. Strains were arranged based on SNPs analysis. Black shaded box indicates gene is present. For *fim*H, numbers within shaded cells, indicate allelic variants. Asterisk within isolates harbouring microcin genes indicates strains had Col replicon.

We used the cut-off value of <28 mm for meropenem (10 μg) reading to screen for carbapenemases as per EUCAST guidelines (www.eucast.org). Twenty-five isolates fulfilled this criterion and were considered putative carbapenemase producers (S2 Fig). Out of them, 22 isolates harboured known carbapenemase genes. Thirteen of them were NDM with four variants of which NDM-5 was the commonest (8/13), followed by NDM-1 (5/13). Seven isolates were OXA-48-like with both OXA-181 (4/7) and OXA-48 (3/7) variants. The remaining two isolates co-produced both enzymes (Fig 3).

In addition, almost all carbapenemase-producing strains had ESBL enzymes (n = 21/22) except OM481, which was an OXA-48 producer only (Fig 3). Overall, 31 isolates produced ESBL enzyme with CTX-M-15 as the most common ESBL-variant (n = 28/31). All isolates were susceptible to colistin, fosfomycin and tigecycline while all OXA-48-like producers were susceptible to ceftazidime-avibactam (S2 Fig).

Three isolates had no known carbapenemase genes, but meropenem reading <28 mm. They harboured *bla*_AmpC_ (OM112), *bla*_ESBL_ (OM211), or a combination of both genes (OM5639). They were negative by Carba NP, whilst were identified as OXA-48-like or ESBL with porin loss in ROSCO disk (S2 Fig).

Aside from the presence of carbapenemase genes, loss of porins or alteration in AcrAB-TolC efflux pump play a role in CR-EC. Thus, we further looked into chromosomal changes in the three strains which harboured no known carbapenemase genes but were carbapenem non-susceptible. OM5639 had a single variation in *omp*C whereas OM211 had genetic variations in both *omp*F and *omp*C genes. Interestingly, OM112 had a wild-type sequence in *omp*F, *omp*C and *omp*R. Regarding genes regulating the AcrAB-TolC efflux pump, no variation could be seen neither in OM5639 nor in OM112. Yet, we detected one missense mutation in AcrR resulting in a frameshift of valine amino acid at codon 29 (V29fs) in OM211 (S3 Table). The data show, at least in one strain (OM112) the reduced susceptibility to carbapenem could not be attributed neither to carbapenemases nor to mutations in chromosomal genes.

For the rest of the strains, we reported high frequency of missense mutations in porins (OmpC and OmpF), while only one was seen in regulatory OmpR protein in one strain (OM150) (S3 Table). Regarding efflux pump, we reported already known missense mutations in AcrR (T5N), MarR (G103S, Y137H, K62R, S3N), SoxR (T38S, G74R), and SoxS (A12S) to induce overexpression of the AcrAB-TolC efflux pump and contribute to fluoroquinolones and tigecycline resistance in *E*. *coli* [50–55]. Also, we saw a A111T missense mutation in SoxR, which has unclear clinical significance, as it has been seen previously in isolates with normal expression of *sox*S mRNA [52, 55]. Another genetic alteration commonly seen was a frame shift at codon 29 of AcrR regulator protein (V29fs). In the same codon, V29G was reported by others [55]. Additionally, we detected other SNPs that might be significant, but have not been reported previously in the literature (S3 Table).

With regards to the 10 isolates with meropenem zone diameter ≥28 mm, 9 strains carried β-lactamase genes—either *bla*_AmpC_, *bla*_ESBL_, or both. One isolate (OM333), was susceptible to cephalosporins by disk diffusion and lacked any known acquired resistance gene in all screened classes of antibiotics (Fig 3 and S2 Fig).

### Plasmidome and mobile genetic element

Carbapenemase genes are often carried by plasmids. Here, we used *in silico* plasmid replicon (rep) typing approach to reflect on plasmidome content (Table 1). A total of 136 replicons were detected within 34 isolates, with one isolate being plasmid-free (OM1433).

As expected repIncFII (also designated as repIncFIIA) alone or in combination with repIncFIA or/and repIncFIB, was the most common replicon in *E*. *coli* (Table 1) asit was seen in 30 strains. While we detected repIncFIA and repIncFIB in 23 isolates each. Replicon subtyping showed higher diversity in repIncFIB with 7 subtypes, compared to repIncFIA having only 3 subtypes (S2 Fig). Additionally, we detected 4 replicons of colicingenic plasmids (ColBS512, ColKP3, ColpVC, Col156) which usually carry colicin as an important virulence factor in *E*. *coli*.

Usually mobile genetic elements (MGEs) flank ARGs. So we sought to screen for MGEs in close proximity to carbapenemase genes. Out of 22 isolates producing carbapenemase, we could detect MGEs in only five. OM561 had 3 MGEs sandwiched between *bla*_NDM-7_ (IS*5*: *bla*_NDM-7_:IS*26*:IS*Kox3*). Both OM1071 and OM1273 had IS*Kpn19* within the same contig where carbapenemase genes were found. Moreover, OM1301 harboured two carbapenemase genes in two distinct contigs. While the contig with *bla*_OXA-181_ had 2 MGEs (IS*Kpn19*: IS*Kox3*), we could see one MGE (IS*26*) close to *bla*_NDM-5_. Also, IS*Aba14* was seen close to *bla*_NDM-1_ in OM1136. The rest of carbapenemase genes might have MGEs, but this could not be confirmed due to the limitations of short-read sequences.

### Virulence determinants

One of the factors that determines the ability of CR-EC to establish an infection is its arsenal of virulence genes. Here, the total number of virulence genes were 380 ranging from 4 to 29 (Fig 3). OM333 had the highest number of of virulence genes. It belonged to B2-phylogroup and did not have any ARGs (Fig 3). Yet, the lowest number of virulence genes (n = 4) was seen in OM1273 and OM1301 strains. They belonged to phylogroup C and ST410. The virulence factors could be categorized into adhesin, toxin, siderophore and protectin related genes (Table 1).

Among adhesin-related genes, type 1 fimbrin D-mannose specific adhesin protein encoded by *fim*H gene is an important typing scheme in *E*. *coli*. Twelve types of *fim*H were seen in 24 isolates. Six of them were singletons whereas *fim*H54 was the commonest (n = 5). The notorious *fim*H30 (n = 3) and *fim*H64 (n = 1) alleles were detected mainly in B2-strains. Also, *fim*H24 was reported in three isolates belonged to phylogroup C. Eleven isolates could not be typed (Fig 3).

Among toxin-related gene, bacteriocins (colicin and microcin) are known to be produced by *E*. *coli* against competitors as a defence mechanism. Genes codify colicin (*cma*, *cea*, *cia*, *cib*) were detected in 5 isolates whereas those codify microcin (*mch*BCF, *mcm*A and *cva*C) were detected in 4 strains (Fig 3). Col plasmids usually carry bacteriocin genes. Here both repCol and bacteriocin genes were seen together in two isolates (Fig 3). Also, two strains harboured *clb*B (colibactin) which is another toxin-related gene. They belonged to B2 phylogroup (OM333 and OM1692). Besides, all B2-strains (n = 4) carried genes (*pic*, *vat*, *sat*) encoding serine protease autotransporters of Enterobacteriaceae (SPATE) toxins.

Siderophores were common in the studied strains. Aerobactin (*iuc*C, *iut*A) were detected in 12 strains whereas yersinabactin (*irp*2, *fyu*A) were seen in 17 strains. Both aerobactin and yersinabactin were found in 9 isolates. Additionally, salmochelin (*iro*N) was detected in two strains both of them belonged to B2 phylogroup. Moreover, iron transport gene (*sit*A) and iron receptor gene (*chu*A) were detected in 20 and 15 strains, respectively (Fig 3).

Protectin factors include genes related to capsule and those involved in resistance to substances that are unfavourable to bacterial growth. The commonest were *gad* (glutamic decarboxylase) and *ter*C (tellurium iron resistance), as they were seen in most of the strains (n = 34). Another important protectin factor is *iss* (increased serum resistance) which was detected in 21 isolates (60%) and our data show it could be gained by HGT from prophages.

### Prophages carried virulence genes

As phages were used historically to type bacteria, we typed the prophage content *in silico*. In total 200 prophages were detected in 35 isolates. Based on their identity score, 80 were intact, 24 were questionable and 96 were incomplete (S2 Fig). Interestingly, we identified prophages carrying virulence genes (Table 2). For example, the *iss* gene was detected within prophages in 14 isolates, while *irp*2, *kps*E and *kps*MII were detected within prophages in one strain. Noteworthy, most of the prophages harbouring virulence genes were intact (n = 10) (Table 2).

**Table 2:** Virulence genes found within propahges in the studied strains.

Strain	Genes (Identity)	Contigs number	Position in WGS	Position in prophage	Prophage completeness
OM78	*iss* (100%)	3	15367–15660	76–39772	questionable
OM126	*iss* (99.7%)	36	15378–15719	54–16050	intact
OM260	*iss* (100%)	14	5237–5578	88–28717	incomplete
OM347	*iss* (100%)	20	75350–75643	55542–76554	incomplete
OM693	*iss* (99.7%)	26	38970–69812	38970–69566	intact
OM853	*iss* (98.6%)	34	36367–36708	12683–40942	incomplete
OM855	*iss* (99.7%)	43	4609–4950	59–17951	intact
OM898	*iss* (99.7%)	22	56574–56915	26075–77569	intact
OM979	*iss* (99.7%)	21	22639–22980	50–40467	intact
OM1071	*iss* (99.4%)	21	56597–56938	26098–77591	intact
OM1168	*iss* (99.7%)	61	1548–1889	2–16882	intact
OM1341	*iss* (100%)	50	866–1159	2–16038	incomplete
OM1626	*iss* (99.7%)	47	66–17958	66–17958	intact
OM1692	*irp*2 (99.9%)	2	84621–118851	84621–118851	intact
	*kps*E (100%	3	111–4554		questionable
	*kps*MII (100%)				
OM5639	*Iss* (99.7%)	22	56469–56810	38970–73781	intact

### High-risk clones

The spread of resistance genes globally, is linked to particular clones known as high-risk clones (HiRC). Here, we detected three HiRCs among five strains. All were resistant to fluoroquinolones (R) and produced CTX-M-15 (x). Two of them belonged to the virulent B2 phylogroup whereas the remaining three strains belonged to C phylogroup. All of them harboured resistance genes to most classes of antibiotics except colistin, fosfomycin and tigecycline (S2 Fig).

One of the B2-strain was ST131-H30Rx/C2 (OM260). It was resistant to fluoroquinolones due to mutations in GyrA (S83L, D87N) and ParC (S80I, E84V) proteins. It produced AmpC and CTX-M-15 enzymes (S4 Table). The second B2-strain was ST1193-H64Rx (OM1136). It had mutations known to cause fluoroquinolones resistance in GyrA (S83L, D87N), ParC (S80I) and ParE (L416F) proteins. Additionally, it produced CTX-M-15, AmpC and worryingly NDM-1 (S4 Table).

The third clone was ST410-H24RxC which was seen in 3 strains of C phylogroup. All of them carried three repIncF (repIncFII, repIncFIB, repIncFIA) and had repIncX3. Two of them (OM1273 and OM1301) had identical virulence genes (*gad*, *Ipf*A, *ter*C) and identical repIncF with pMLST (F1:A1:B49). In addition to *bla*_CTX-M-15_, both strains possessed *bla*_TEM1B_*-bla*_OXA1_-*str*AB*-dfr*A17*-sul*2*-tet*B resistance genes. Yet, OM1273 harboured *bla*_OXA-181_ only whereas OM1301 co-harboured *bla*_OXA-181_ and *bla*_NDM-5_. They had quite destinct mutations encoding fluoroquinolones resistance (S4 Table). The remaining strain (OM561) carried *bla*_NDM-7_ gene and repIncF with pMLST (F31:A4: B1). It did not have any mutations in ParC and ParE proteins. Yet, we saw two missense mutations in GyrA (S83L, D87N) (S4 Table).

### Correlating genomic and clinical epidemiologies

To achieve a better understanding of the spread of CR-EC in Oman, we assessed potential transmission particularly for strains with ≤ 10 SNPs apart. There were 8 pairs with this criterion (Table 3). Interestingly, one pair (OM79 and OM147) was isolated within a month from the same neonatal patient with a travel history to India (S2 Table). Despite both were NDM-producers, OM79 carried NDM-4 variant whereas OM147 had NDM-1 (Fig 3). Nevertheless, both strains most likely arose from the same ancestor, apparently their contents of accessory genes evolved. Whether the alteration occurred within the host who might encounter the strain initially from India yet due to change in antibiotic pressure, the strain content of ARGs changed or the patient might encounter two independent strains circulating in the hospital is not clear from the data we had. Generally, if the isolates carry different carbapenemase genes they are unlikely to be involved in the same transmission event despite their similar genetic background. Hence, we will focus on pairs with the same carbapenemase genes as a potential transmission events.

**Table 3:** Comparison of typing approaches and their clinical relatedness for isolates with ≤ 10 SNPs apart.

Strain 1	Strain 2	Isolation gap (Month)	Source (Hospital)	Carbapenemase genes	SNPs (25,534)	wgMLST (25,000)	cgMLST (2,500)	MLST (7)
OM852	OM898	1	same	same	0	2	1	0
OM855	OM1626	4	different	same	1	18	7	0
OM79*	OM147*	1	same	different	2	27	7	0
OM839	OM82	0	same	no^‡^	7	347	191	0
OM1301	OM1273	0	different	similar^§^	8	85	25	0
OM855	OM1071	1	same	different	9	215	87	0
OM1626	OM1071	3	same	different	10	233	80	0
OM347	OM234	1	same	different	10	126	68	0

Clusters were arranged ascendingly based on the number of core SNPs between them. Number within parentheses indicates the total number of SNPs/genes used in each scheme.

*Isolates were obtained from the same patient.

^‡^ Isolates produced CTX-M-15.

^§^OM1301 harboured *bla*_NDM-5_ and *bla*_OXA-181_, while OM1273 only had *bla*_OXA-181_.

The first event involved two ST1702 isolates (OM852 and OM898) with no SNPs differences. They were separated by one and two genes with cgMLST and wgMLST, respectively (Table 3). The two isolates had the same accessory genes as well as identical replicon types of repIncF pMLST (F2:A4:B-) and IncI1 pMLST(C) (S2 Fig). They were isolated one month apart from the same hospital, yet in different wards, at least at the time point of sampling (S2 Table). This suggest local transmission, although infection control data to support the likelihood of such an event could not be obtained.

The second possible transmission event involved two strains of ST167 (OM855, OM1626). They had the same NDM variant (NDM-5) and pMLST (F36:A4:B-). OM855 was isolated first in a screening sample from a Yemeni patient who was admitted at Khoula hospital (Muscat) on May and discharged four days later. He was paraplegic for a month which could suggest another medical treatment was involved prior to this admission (S2 Table). In September, OM1626 was isolated in a clinical sample (wound) obtained from an Omani patient who was admitted at SQH hospital in Salalah. They had identical accessory genes including the same IncF replicon (F36:A14:B-). Despite the close genetic similarity between them, there was no clear temporospatial link between the individuals to infer a common source of infection. Since Yemen is close to Salalah, we speculate the first patient could be a potential carrier for the ancestor strain who might initially received a medical care at SQH (Salalah) prior to his admission at Khoula hospital (Muscat) and became a carrier to the clone isolated from SQH four months later (OM1626). However, it is not clear from the patient history whether he was admitted at SQH previously.

We expected that patients’ citizenship or travel history could correlate with the occurrence of certain resistance genes. Interestingly, the isolates from Indian patients did not produce NDM nor OXA-48-like enzymes. Data on foreign travel was available only for 10 patients (29%), hence no proper statistical analysis could be carried out (Table 1).

On the other side, out of five deceased patients two of them had bacteraemia (OM333, OM78), four had history of diabetes mellitus and hypertension (OM78, OM150, OM333, OM693), one had gastrointestinal bleeding (OM82) and one had kidney graft dysfunction (OM693). Thus, we cannot exclude that the deaths were attributed to non-infectious co-morbidities (S2 Table). Notably, one of the expired patients had a strain with no acquired resistance genes but with the highest number of virulence genes (OM333). It belonged to B2 lineage and ST73 (Fig 3).

## Discussion

The data presented herein stemmed from a genomic epidemiology and surveillance study of CR-EC from Oman in 2015. We saw a polyclonal population structure as multiple STs were seen with low frequency. We demonstrate that all confirmed carbapenemase-producing *E*. *coli* from Oman belonged to either NDM (59%) or OXA-48-like (32%) classes, or a combination (9%). The high-risk subclones, ST131-H30Rx/C2, ST410-H24RxC and ST1193-H64RxC were detected. The latter was associated with NDM, to our knowledge for the first time in international literature.

Over the last decade, WGS has been increasingly used for epidemological typing, and multiple online pipelines are available for WGS analyses [26, 56, 57]. While this is an advantage it does create challenges yet to be resolved including, but not limited to, how to correlate studies utilising different approaches. Another challenge is selecting the approach with optimal resolution for the aim of the study whether surveillance or tracing the source of an outbreak, considering the pros and cons in each approach. A third challenge is the management of recombination, as most of the approaches rely on the recognition of sites with high SNP density, which is not distinguishable from region with high rate of heterogenisity [58]. Here, we thought to mask the 7 house-keeping genes prior to variant calling and filtering out recombination sites, to eliminate the risk of technical error, as the collection is genetically diverse. We saw good alignment between SNP-calling and cgMLST scheme in allelic differences. We reported eight putative transmission pairs based on SNPs data (Table 3), although some of them were less likely, due to for instance presence of different carbapenemase genes. The lack of clinical data supporting some of the episodes questions whether the algorithm for removal of recombination could be too strict, thus increasing the overall resolution falsely [59]. Moreover, the downstream analyses are heavily impacted by the quality of reads and the assembled genome used as input.

We detected herein a polyclonal population with diverse sequence types of CR-EC in Oman. Twenty-one STs were seen in 35 isolates yet most of them were singletons. The most commonly encountered ST was ST38, belonging to phylogroup D (Fig 1). We also detected ST410-H24RxC in three strains, a subclone that emerged recently [60, 61]. ST410 has been so far associated with the production of OXA-181 and/or NDM-5 carried on IncX3 plasmid. Both carbapenemases were detected in one strain. Yet, only NDM-5 or NDM-7 were detected in the remaining strains (Fig 3). Due to the fact that only short reads are available, we were unable to determine whether carbapenemase genes were located on IncX3 or on another plasmid, but we could confirm the presence of repIncX3 in the three strains (S4 Table). ST410 with NDM-7 has been reported from China previously [62]. Noteworthy, the three strains in our data belonged to phylogroup C rather than A. The latter was reported for ST410-H24RxC in literature [61].

Furthermore, HiRC ST131-H30Rx/C2 (OM260) and ST1193-H64Rx (OM1136) were detected. The latter carried *bla*_NDM-1_. To our knowledge this is the first report of ST1193-H65Rx with the *bla*_NDM_ gene, now named ST1193H65RxC where C indicates the presence of carbapenemase gene. Recently, two strains with ST1193 and ST131 were reported to carry *bla*_IMP_ gene from Japan. However, no data were available at subclonal level [63]. ST131-H30Rx/C2, was reported recently with different carbapenemases from UK [18]. Such HiRCs with resistance and virulence genes are worrisome and concerning globally due to their rapid dissemination with CTX-M-15 previously and being of B2 phylogroup.

The virulent B2 phylogroup was detected in four isolates. None of them but OM1136, described above as ST1193-H64RxC, harboured carbapenemase genes. It had *bla*_NDM-1_ and IS*Aba14* within the same contig. Interestingly, it had the second highest number of ARGs (n = 18), only one gene less than OM1301 (n = 19). To the contrary, another B2-strain had no ARGs for all screened classes of antibiotics (OM333-ST73-O6:H1) but had the highest number of virulence genes (n = 12) and only repIncX1 (Fig 3 and S2 Fig). The strain was isolated from the bloodstream of a patient who eventually expired (S2 Table). ST73-O6:H1-B2 clone was reported with CTX-M-15 from Egypt and Japan previously but not with carbapenemases so far to our knowledge [64, 65]. It is a notorious clone due to its association with bloodstream infection and expression of a high number of virulence genes [66]. Worth noting, all B2-strains lacked a CRISPR system (Fig 2), corroborating previous reports [67, 68]. The absence of CRISPR in the B2 lineage likely increases their susceptibility to acquisition of mobile genetic elements.

Despite CRISPR system in *E*. *coli* known to be static, it could be a valuable tool to recognise outlier strains, perhaps to a higher degree than for instance MLST [68]. Here, OM664 showed the highest number of spacers (n = 46) which were unique (Fig 2). This highly divergent strain was isolated from fecal screening in a patient with travel history to India (S2 Table). The strain belonged to ST488, a rare ST reported from India previously [69]. This suggests the strain was recently introduced to Oman from India and not yet merged genetically with other local strains, hence all spacers were unique. It had *bla*_NDM-5_ which was reported recently from India and China [70, 71].

Likewise, we found NDM-5 as the most frequent variant of NDM in this collection (n = 8). Also, we detected NDM-7 in one isolate which mirrors a report of low prevalence of NDM-7 in the Arabian Peninsula [72]. In this dataset, NDM and OXA-48-like enzymes were the only carbapenemases in CR-EC from Oman. The resistance pattern we saw in this study echoes previous reports from Oman. For example, the first report of CR-EC from Oman was in 2012 from the Royal Hospital in Muscat, with four strains featuring NDM-1 and OXA-48-like enzymes [73]. Additionally, from the same hospital, Sonnevend et al., examined ten *E*. *coli* strains and reported the presence of *bla*_NDM-7_ in one of them in 2015 [74].

Beside the gain of new genes encodying carbapenemases, alteration in already existing genes could yield a CR-EC phenotype. Despite there was no porin loss in all isolates, we noticed genetic variations in *omp*F, *omp*C and *omp*R genes, none of these mutations were published previously (S3 Table). Regarding AcrAB-TolC efflux pump, we found genetic alterations in all regulating genes with *sox*S and *mar*A having the lowest variation in only one isolate for each gene. Further experimental work is required to verify their impact. Noteworthy, five isolates (OM112, OM839, OM1301, OM853, OM979) showed wild-type in all screened genes (S3 Table).

As expected, isolates with B2 phylogroup were the most virulent strains. All of them (n = 4) harboured genes (*pic*, *vat*, *sat*) codify for SPATE toxins. They were isolated from blood (OM3333-ST73; *pic*, *sat*, *vat*), urine (OM1692-ST127; *vat*, *pic*), tracheal secretion (OM1136-ST1193; *sat*, *vat*) and biopsy (OM260-ST131; *sat*). Interestingly, *pic* (protease involved in intestinal colonization) and *sat* (secreted autotransporter toxin) but not *vat* (vacuolating autotransporter toxin) were linked to Enteroaggregative *E*. *coli* (EAEC) previously [75, 76]. Besides, Pic implicates mucinase activity, serum resistance, hemagglutination and promote intestinal colonisation [77]. While Sat and Vat are known to cause cytotoxic damages and were associated with uropathogenic *E*. *coli* (UPAC) or urosepsis clinical isolates previously [78–80]. Generaly, most of the detected virulence genes were reported previously in extraintestinal pathogenic *E*. *coli* (ExPEC). For example, *fim*H, *iha*, *sfa*, *ast*A, *iro*N, *cnf*1, *sat*, *iss* and *irp* [81–83].

Another important virulence factor is the presence of siderophore which plays a role in iron acquisition. Here, we reported three siderophore systems (aerobactin, yersiniabactin, salmochelin) in more than half of the studied strains (n = 19). No enterobactin related genes were detected despite being the most efficient system in scavening iron [84]. The least common was salmochelin as it was found in two isolates of B2 phylogroup. Interestingly, one of them (OM1692) was isolated from urine as Watts et al., suggested that salmochelin plays a role in urinary tract colonisation [84].

Our data support that prophages could act as a vehicle for virulence genes including *iss* (Table 2). Studies of *iss* evolution in *E*. *coli* have demonstrated similarities with the *bor* gene from lambda (λ) bacteriophage [62]. With regards to ARGs, we did not detect any ARGs within prophages. Yet, there were reports supporting prophages could carry ARGs. For example, *bla*_CTX-M-27_ was detected in SJ46 (P1-like bacteriophage) from *Salmonella spp* in China [85]. Prophages with *bla*_TEM_, *bla*_CTX-M_ and *qnr*S have been reported previously [4–6]. Interestingly, Kleinheinz et al., detected *bla*_OXA-48-like_ from a prophage *in silico* [86]. However, a recent analysis of 659 putative prophages in the human gut revealed that only three prophages (0.46%) carried resistance genes [87]. The role of prophages in the spread of resistance genes is still largely unclear and remains to be further investigated.

On the other hand, plasmid is known to carry resistance genes and spread them horizontally. However, we reported a strain (OM1433) with no plasmid replicon but it featured *bla*_OXA-48-like_ which is commonly linked to IncL/M (pOXA48) plasmid (S2 Fig). The strain belonged to ST38 and was umbilicus isolate of an infant with preterm birth (S2 Table). Chromosomally integrated *bla*_OXA-48_ has been reported previously in ST38, but we could not exclude the gene might be located on an untypable plasmid [88–90].

The study has some limitations. One of them is the lack of a control group of wild-type isolates from the same time-period. It would be of interest, for example, to compare CRISPR-cas system and prophage profiles in susceptible and resistant strains. Besides, the CR-EC isolates in this study were relatively few even though it presents the largest collection of CR-EC from Oman during a period of 10 months. In future studies the duration could be increased over a year. Another improvement would be to apply long-read sequencing, enabling more detailed analysis of plasmid content, instead of the replicon approach implemented here.

The primary significance of the study resides in being the first to profile clonal diversity, replicons and virulence contents for EC-CR and -ESBL from Oman at a subnational level involving multiple centres. Importantly, we report ST1193-H64RxC with *bla*_NDM_, to our knowledge for the first time. A close monitoring of such HiRC is warranted to curb major transmission events at an early stage.

## Supporting information

S1 Fig Map of Arabian Peninsula including Oman and the geographical locations of hospitals where samples were obtained. The 35 samples were obtained from 8 tertiary hospitals in Oman. Four of them are located in the capital, Muscat. Buraimi Hospital is located at the border with United Arab Emirates (UAE) whereas Sultan Qaboos Hospital (SQH) in Salalah is located near Yemen. Names of the cities in Oman’s map represented with bold font whereas hospitals’ names were listed below them. The number of samples obtained from each hospitals was represented within parenthesis.
(PDF)

S2 Fig Heatmap for the analysis of genomic contents and phenotypic readings. Heatmap shows phenotypic readings, ARGs in antibiotic classes other than β-lactam, plasmid replicons and prophages for the studied 35 strains.
(PDF)

S1 Table Genomic quality of sequenced strains and their corresponding SRA accession numbers. (PDF)

S2 Table Demographic data. (PDF)

S3 Table Genetic alterations in regulatory proteins for porins and AcrAB-TolC efflux pump. (PDF)

S4 Table High-risk subclones identified in the studied strains. (PDF)

## References

[1] Vila J., Sáez-López E., Johnson J.R., Römling U., Dobrindt U., Cantón R., et al, *Escherichia coli*: an old friend with new tidings. FEMS Microbiol Rev, 2016 40(4): p. 437–463. 10.1093/femsre/fuw005 28201713

[2] Levy Hara G., Gould I., Endimiani A., Pardo P.R., Daikos G., Hsueh P.R., et al, Detection, treatment, and prevention of carbapenemase-producing Enterobacteriaceae: recommendations from an International Working Group. J Chemother, 2013 25(3): p. 129–40. 10.1179/1973947812Y.0000000062 23783137

[3] Nordmann P., Dortet L., and Poirel L., Carbapenem resistance in Enterobacteriaceae: here is the storm! Trends Mol Med 2012 18(5): p. 263–72. 10.1016/j.molmed.2012.03.003 22480775

[4] Brown-Jaque M., Rodriguez Oyarzun L., Cornejo-Sánchez T., Martín-Gómez M.T., Gartner S., de Gracia J., et al, Detection of Bacteriophage Particles Containing Antibiotic Resistance Genes in the Sputum of Cystic Fibrosis Patients. Frontiers in microbiology, 2018 9: p. 856–856. 10.3389/fmicb.2018.00856 29765367 PMC5938348

[5] Colomer-Lluch M., Jofre J., and Muniesa M., Antibiotic resistance genes in the bacteriophage DNA fraction of environmental samples. PLoS One, 2011 6(3): p. e17549 10.1371/journal.pone.0017549 21390233 PMC3048399

[6] Colomer-Lluch M., Jofre J., and Muniesa M., Quinolone resistance genes (*qnrA* and *qnrS*) in bacteriophage particles from wastewater samples and the effect of inducing agents on packaged antibiotic resistance genes. J Antimicrob Chemother, 2014 69(5): p. 1265–74. 10.1093/jac/dkt528 24458509

[7] Enault F., Briet A., Bouteille L., Roux S.A.-O., Sullivan M.B., and Petit M.A., Phages rarely encode antibiotic resistance genes: a cautionary tale for virome analyses. Nucleic Acids Research, 2018 46(W1): p. W246–W251. 27326545 10.1038/ismej.2016.90 PMC5315482

[8] Parsley L.C., Consuegra E.J., Kakirde K.S., Land A.M., Harper W.F., and Liles M.R., Identification of Diverse Antimicrobial Resistance Determinants Carried on Bacterial, Plasmid, or Viral Metagenomes from an Activated Sludge Microbial Assemblage. Applied and Environmental Microbiology, 2010 76(11): p. 3753 10.1128/AEM.03080-09 20382816 PMC2876469

[9] Barrangou R., Fremaux C., Deveau H., Richards M., Boyaval P., Moineau S., et al, CRISPR provides acquired resistance against viruses in prokaryotes. Science, 2007 315(5819): p. 1709–12. 10.1126/science.1138140 17379808

[10] Shariat N. and Dudley E.G., CRISPRs: molecular signatures used for pathogen subtyping. Appl Environ Microbiol, 2014 80(2): p. 430–9. 10.1128/AEM.02790-13 24162568 PMC3911090

[11] Barrangou R., Fremaux C., Deveau H., Richards M., Boyaval P., Moineau S., et al, CRISPR Provides Acquired Resistance Against Viruses in Prokaryotes. Science, 2007 315(5819): p. 1709 10.1126/science.1138140 17379808

[12] Dang T.N., Zhang L., Zollner S., Srinivasan U., Abbas K., Marrs C.F., et al, Uropathogenic *Escherichia coli* are less likely than paired fecal *E*. *coli* to have CRISPR loci. Infect Genet Evol, 2013 19: p. 212–8. 10.1016/j.meegid.2013.07.017 23891665

[13] Delannoy S., Beutin L., and Fach P., Use of clustered regularly interspaced short palindromic repeat sequence polymorphisms for specific detection of enterohemorrhagic *Escherichia coli* strains of serotypes O26:H11, O45:H2, O103:H2, O111:H8, O121:H19, O145:H28, and O157:H7 by real-time PCR. J Clin Microbiol, 2012 50(12): p. 4035–40. 10.1128/JCM.02097-12 23035199 PMC3503007

[14] Louwen R., Staals R.H., Endtz H.P., van Baarlen P., and van der Oost J., The role of CRISPR-Cas systems in virulence of pathogenic bacteria. Microbiol Mol Biol Rev, 2014 78(1): p. 74–88. 10.1128/MMBR.00039-13 24600041 PMC3957734

[15] Yin S., Jensen M.A., Bai J., Debroy C., Barrangou R., and Dudley E.G., The evolutionary divergence of Shiga toxin-producing *Escherichia coli* is reflected in clustered regularly interspaced short palindromic repeat (CRISPR) spacer composition. Appl Environ Microbiol, 2013 79(18): p. 5710–20. 10.1128/AEM.00950-13 23851088 PMC3754162

[16] Woodford N., Turton J.F., and Livermore D.M., Multiresistant Gram-negative bacteria: the role of high-risk clones in the dissemination of antibiotic resistance. FEMS Microbiol Rev, 2011 35(5): p. 736–55. 10.1111/j.1574-6976.2011.00268.x 21303394

[17] Pitout J.D. and DeVinney R., *Escherichia coli* ST131: a multidrug-resistant clone primed for global domination. F1000Res, 2017 6. 10.12688/f1000research.10609.1 PMC5333602 28344773

[18] Ellaby N., Doumith M., Hopkins K.L., Woodford N., and Ellington M.J., Emergence of diversity in carbapenemase-producing *Escherichia coli* ST131, England, January 2014 to June 2016. Eurosurveillance, 2019 24(37): p. 1800627. 10.2807/1560-7917.ES.2019.24.37.1800627 PMC6749775 31530344

[19] Johnson T.J., Elnekave E., Miller E.A., Munoz-Aguayo J., Flores Figueroa C., Johnston B., et al, Phylogenomic Analysis of Extraintestinal Pathogenic *Escherichia coli* Sequence Type 1193, an Emerging Multidrug-Resistant Clonal Group. Antimicrobial Agents and Chemotherapy, 2019 63(1): p. e01913–18. 10.1128/AAC.01913-18 30348668 PMC6325179

[20] Minsitry of Health. Oman Antimicrobial Resistance Surveillance System Annual Report. 2017. Accessed 20 March 2020; https://www.moh.gov.om/documents/236878/0/OMASS+2017/ca052ace-691a-1919-7b96-41f79539660a.

[21] The Centers for Disease Control and Prevention (CDC), Facility Guidance for Control of Carbapenem-resistant Enterobacteriaceae (CRE) November 2015 Update. 2015.

[22] Ministry of Health. Oman Antimicrobial Resistance Surveillance System Annual Report. 2018. Accessed 21 March 2020.

[23] The Center for Disease, D.E.P. ResistanceMap: Antibiotic resistance. 2020 20 March 2020.

[24] European Committee on Antimicrobial Susceptibility Testing (EUCAST), Breakpoints tables for interpretation of MICs and zone diameters. 2019.

[25] Poirel L. and Nordmann P., Rapidec Carba NP Test for Rapid Detection of Carbapenemase Producers. Journal of Clinical Microbiology, 2015 53(9): p. 3003 10.1128/JCM.00977-15 26085619 PMC4540946

[26] Zhou Z., Alikhan N.-F., Mohamed K., Fan Y., Agama Study G., and Achtman M., The EnteroBase user's guide, with case studies on Salmonella transmissions, Yersinia pestis phylogeny, and Escherichia core genomic diversity. Genome research, 2020 30(1): p. 138–152. 10.1101/gr.251678.119 31809257 PMC6961584

[27] H, L., Aligning sequence reads, clone sequences and assembly contigs with BWA-MEM. 266. arXiv:1303.3997v1. [q-bio.GN]. 267. 2013.

[28] Li H., Handsaker B., Wysoker A., Fennell T., Ruan J., Homer N., et al, The Sequence Alignment/Map format and SAMtools. Bioinformatics, 2009 25(16): p. 2078–9. 10.1093/bioinformatics/btp352 19505943 PMC2723002

[29] Walker B.J., Abeel T., Shea T., Priest M., Abouelliel A., Sakthikumar S., et al, Pilon: An Integrated Tool for Comprehensive Microbial Variant Detection and Genome Assembly Improvement. PLOS ONE, 2014 9(11): p. e112963 10.1371/journal.pone.0112963 25409509 PMC4237348

[30] Challis, R., rjchallis/assembly-stats. Zenodo. 10.5281/zenodo.322347. 2017

[31] Alikhan Nf, Z Z., Sergeant Mj and Achtman M., A genomic overview of the population structure of *Salmonella*. PLoS Genet, 2018 14(4): p. e1007261 10.1371/journal.pgen.1007261 29621240 PMC5886390

[32] Beghain J., Bridier-Nahmias A., Le Nagard H., Denamur E., and Clermont O., ClermonTyping: an easy-to-use and accurate *in silico* method for *Escherichia* genus strain phylotyping. Microbial genomics, 2018 4(7): p. e000192. 10.1099/mgen.0.000192 PMC6113867 29916797

[33] T, S., snippy: fast bacterial variant calling from NGS reads. 2015.

[34] Croucher N.J., Page A.J., Connor T.R., Delaney A.J., Keane J.A., Bentley S.D., et al, Rapid phylogenetic analysis of large samples of recombinant bacterial whole genome sequences using Gubbins. Nucleic acids research, 2015 43(3): p. e15 10.1093/nar/gku1196 25414349 PMC4330336

[35] Page A.J., Taylor B., Delaney A.J., Soares J., Seemann T., Keane J.A., et al, SNP-sites: rapid efficient extraction of SNPs from multi-FASTA alignments. Microbial genomics, 2016 2(4): p. e000056–e000056. 10.1099/mgen.0.000056 28348851 PMC5320690

[36] Stamatakis A., RAxML version 8: a tool for phylogenetic analysis and post-analysis of large phylogenies. Bioinformatics, 2014 30(9): p. 1312–1313. 10.1093/bioinformatics/btu033 24451623 PMC3998144

[37] Letunic, I. and P. Bork, Interactive Tree Of Life (iTOL) v4: recent updates and new developments. 2019. 10.1093/nar/gkz239 PMC6602468 30931475

[38] Schürch A.C., Arredondo-Alonso S., Willems R.J.L., and Goering R.V., Whole genome sequencing options for bacterial strain typing and epidemiologic analysis based on single nucleotide polymorphism versus gene-by-gene based approaches. Clinical Microbiology and Infection, 2018 24(4): p. 350–354. 10.1016/j.cmi.2017.12.016 29309930

[39] Couvin D., Bernheim A., Toffano-Nioche C., Touchon M., Michalik J., Neron B., et al, CRISPRCasFinder, an update of CRISRFinder, includes a portable version, enhanced performance and integrates search for Cas proteins. Nucleic Acids Res 2018 46(W1): p. W246–W251. 10.1093/nar/gky425 29790974 PMC6030898

[40] Grissa I., Vergnaud G., and Pourcel C., CRISPRFinder: a web tool to identify clustered regularly interspaced short palindromic repeats. Nucleic Acids Res, 2007 35: p. W52–7. 10.1093/nar/gkm360 17537822 PMC1933234

[41] Zhang F., Zhao S., Ren C., Zhu Y., Zhou H., Lai Y., et al, CRISPRminer is a knowledge base for exploring CRISPR-Cas systems in microbe and phage interactions. Commun Biol, 2018 1: p. 180 10.1038/s42003-018-0184-6 30393777 PMC6208339

[42] Zankari E., Hasman H., Cosentino S., Vestergaard M., Rasmussen S., Lund O., et al, Identification of acquired antimicrobial resistance genes. J Antimicrob Chemother, 2012 67(11): p. 2640–4. 10.1093/jac/dks261 22782487 PMC3468078

[43] Carattoli A., Bertini A., Villa L., Falbo V., Hopkins K.L., and Threlfall E.J., Identification of plasmids by PCR-based replicon typing. J Microbiol Methods, 2005 63(3): p. 219–28. 10.1016/j.mimet.2005.03.018 15935499

[44] Carattoli A., Zankari E., García-Fernández A., Voldby Larsen M., Lund O., Villa L., et al, In silico detection and typing of plasmids using PlasmidFinder and plasmid multilocus sequence typing. Antimicrobial agents and chemotherapy, 2014 58(7): p. 3895–3903. 10.1128/AAC.02412-14 24777092 PMC4068535

[45] Joensen K.G., Scheutz F., Lund O., Hasman H., Kaas R.S., Nielsen E.M., et al, Real-time whole-genome sequencing for routine typing, surveillance, and outbreak detection of verotoxigenic *Escherichia coli*. J Clin Microbiol, 2014 52(5): p. 1501–10. 10.1128/JCM.03617-13 24574290 PMC3993690

[46] Arndt D., Grant J.R., Marcu A., Sajed T., Pon A., Liang Y., et al, PHASTER: a better, faster version of the PHAST phage search tool. Nucleic Acids Research, 2016 44: p. W16–W21. 10.1093/nar/gkw387 27141966 PMC4987931

[47] Zhou Y., Liang Y., Lynch K.H., Dennis J.J., and Wishart D.S., PHAST: a fast phage search tool. Nucleic Acids Res, 2011 39: p. W347–52. 10.1093/nar/gkr485 21672955 PMC3125810

[48] Kleinheinz K.A., Joensen K.G., and Larsen M.V., Applying the ResFinder and VirulenceFinder web-services for easy identification of acquired antibiotic resistance and *E*. *coli* virulence genes in bacteriophage and prophage nucleotide sequences. Bacteriophage, 2014 4(1): p. e27943–e27943. 10.4161/bact.27943 24575358 PMC3926868

[49] Al-Aamri A.K., Padmadas S.S., Zhang L.C., and Al-Maniri A.A., Disentangling age-gender interactions associated with risks of fatal and non-fatal road traffic injuries in the Sultanate of Oman. B. M. J. Glob Health, 2017 2(3): p. e000394. 10.1136/bmjgh-2017-000394 PMC5623270 29018585

[50] Aly S.A., Boothe D.M., and Suh S.-J., A novel alanine to serine substitution mutation in SoxS induces overexpression of efflux pumps and contributes to multidrug resistance in clinical *Escherichia coli* isolates. Journal of Antimicrobial Chemotherapy, 2015 70(8): p. 2228–2233. 10.1093/jac/dkv105 25921515

[51] Keeney D., Ruzin A., McAleese F., Murphy E., and Bradford P.A., MarA-mediated overexpression of the AcrAB efflux pump results in decreased susceptibility to tigecycline in *Escherichia coli*. J Antimicrob Chemother, 2008 61(1): p. 46–53. 10.1093/jac/dkm397 17967850

[52] Koutsolioutsou A., Peña-Llopis S., and Demple B., Constitutive *sox*R mutations contribute to multiple-antibiotic resistance in clinical *Escherichia coli* isolates. Antimicrobial agents and chemotherapy, 2005 49(7): p. 2746–2752. 10.1128/AAC.49.7.2746-2752.2005 15980345 PMC1168631

[53] Maneewannakul K. and Levy S.B., Identification for mar mutants among quinolone-resistant clinical isolates of *Escherichia coli*. Antimicrobial agents and chemotherapy, 1996 40(7): p. 1695–1698. 10.1128/AAC.40.7.1695 8807064 PMC163397

[54] Webber M.A. and Piddock L.J., Absence of mutations in *mar*RAB or *sox*RS in *acr*B-overexpressing fluoroquinolone-resistant clinical and veterinary isolates of *Escherichia coli*. Antimicrobial agents and chemotherapy, 2001 45(5): p. 1550–1552. 10.1128/AAC.45.5.1550-1552.2001 11302826 PMC90504

[55] Zayed A.A., Essam T.M., Hashem A.G., and El-Tayeb O.M., 'Supermutators' found amongst highly levofloxacin-resistant *E*. *coli* isolates: a rapid protocol for the detection of mutation sites. Emerg Microbes Infect, 2015 4(1): p. e4 10.1038/emi.2015.4 26038761 PMC4317672

[56] Jolley K.A., Bray J.E., and Maiden M.C.J., Open-access bacterial population genomics: BIGSdb software, the PubMLST.org website and their applications. Wellcome open research, 2018 3: p. 124–124. 10.12688/wellcomeopenres.14826.1 30345391 PMC6192448

[57] Ruan Z. and Feng Y., BacWGSTdb, a database for genotyping and source tracking bacterial pathogens. Nucleic Acids Res, 2016 44(D1): p. D682–7. 10.1093/nar/gkv1004 26433226 PMC4702769

[58] Husmeier D., Discriminating between rate heterogeneity and interspecific recombination in DNA sequence alignments with phylogenetic factorial hidden Markov models. Bioinformatics, 2005 21(suppl_2): p. ii166–ii172. 16204097 10.1093/bioinformatics/bti1127

[59] Croucher N.J., Harris S.R., Grad Y.H., and Hanage W.P., Bacterial genomes in epidemiology—present and future. Philos Trans R Soc Lond B Biol Sci, 2013 368(1614): p. 20120202 10.1098/rstb.2012.0202 23382424 PMC3678326

[60] Feng Y., Liu L., Lin J., Ma K., Long H., Wei L., et al, Key evolutionary events in the emergence of a globally disseminated, carbapenem resistant clone in the *Escherichia coli* ST410 lineage. Communications Biology, 2019 2(1): p. 322. 31482141 10.1038/s42003-019-0569-1 PMC6715731

[61] Roer L., Overballe-Petersen S., Hansen F., Schonning K.A.-O.X., Wang M., Roder B.L., et al, *Escherichia coli* Sequence Type 410 Is Causing New International High-Risk Clones. mSphere, 2018 3(4): p. pii: e00337-18. 10.1128/mSphere.00337-18 PMC6052333 30021879

[62] Xu J. and He F., Characterization of a NDM-7 carbapenemase-producing *Escherichia coli* ST410 clinical strain isolated from a urinary tract infection in China. Infect Drug Resist, 2019 12: p. 1555–1564. 10.2147/IDR.S206211 31239731 PMC6559143

[63] Hayakawa K., Nakano R., Hase R., Shimatani M., Kato H., Hasumi J., et al, Comparison between IMP carbapenemase-producing Enterobacteriaceae and non-carbapenemase-producing Enterobacteriaceae: a multicentre prospective study of the clinical and molecular epidemiology of carbapenem-resistant Enterobacteriaceae. Journal of Antimicrobial Chemotherapy, 2019 75(3): p. 697–708. 10.1093/jac/dkz501 31789374

[64] Fam N., Leflon-Guibout V., Fouad S., Aboul-Fadl L., Marcon E., Desouky D., et al, CTX-M-15-Producing Escherichia coli Clinical Isolates in Cairo (Egypt), Including Isolates of Clonal Complex ST10 and Clones ST131, ST73, and ST405 in Both Community and Hospital Settings. Microbial Drug Resistance, 2010 17(1): p. 67–73. 10.1089/mdr.2010.0063 21128836

[65] Suzuki S., Shibata N., Yamane K., Wachino J.-i., Ito K., and Arakawa Y., Change in the prevalence of extended-spectrum-β-lactamase-producing Escherichia coli in Japan by clonal spread. Journal of Antimicrobial Chemotherapy, 2008 63(1): p. 72–79. 10.1093/jac/dkn463 19004839

[66] Riley L.W., Pandemic lineages of extraintestinal pathogenic Escherichia coli. Clinical Microbiology and Infection, 2014 20(5): p. 380–390. 10.1111/1469-0691.12646 24766445

[67] Diez-Villasenor C., Almendros C., Garcia-Martinez J., and Mojica F.J., Diversity of CRISPR loci in *Escherichia coli*. Microbiology, 2010 156(5): p. 1351–1361. 10.1099/mic.0.036046-0 28206910

[68] Touchon M., Charpentier S., Clermont O., Rocha E.P., Denamur E., and Branger C., CRISPR distribution within the *Escherichia coli* species is not suggestive of immunity-associated diversifying selection. J Bacteriol, 2011 193(10): p. 2460–7. 10.1128/JB.01307-10 21421763 PMC3133152

[69] Choudhury N.A., Paul D., Chakravarty A., Bhattacharjee A., and Dhar Chanda D., IncX3 plasmid mediated occurrence of bla(NDM-4) within *Escherichia coli* ST448 from India. J Infect Public Health, 2018 11(1): p. 111–114. 10.1016/j.jiph.2017.06.008 28676284

[70] Devanga Ragupathi N.K., Veeraraghavan B., Muthuirulandi Sethuvel D.P., Anandan S., Vasudevan K., Neeravi A.R., et al, First Indian report on genome-wide comparison of multidrug-resistant *Escherichia coli* from blood stream infections. PLoS One, 2020 15(2): p. e0220428 10.1371/journal.pone.0220428 32101543 PMC7043739

[71] Liang W.-j., Liu H.-y., Duan G.-C., Zhao Y.-x., Chen S.-y., Yang H.-Y., et al, Emergence and mechanism of carbapenem-resistant *Escherichia coli* in Henan, China, 2014. Journal of Infection and Public Health, 2018 11(3): p. 347–351. 10.1016/j.jiph.2017.09.020 29107607

[72] Pal T., Ghazawi A., Darwish D., Villa L., Carattoli A., Hashmey R., et al, Characterization of NDM-7 Carbapenemase-Producing *Escherichia coli* Isolates in the Arabian Peninsula. Microb Drug Resist, 2017 23(7): p. 871–878. 10.1089/mdr.2016.0216 28156193

[73] Dortet L., Poirel L., Al Yaqoubi F., and Nordmann P., NDM-1, OXA-48 and OXA-181 carbapenemase-producing Enterobacteriaceae in Sultanate of Oman. Clin Microbiol Infect, 2012 18(5): p. E144–8. 10.1111/j.1469-0691.2012.03796.x 22404169

[74] Sonnevend A., Ghazawi A.A., Hashmey R., Jamal W., Rotimi V.O., Shibl A.M., et al, Characterization of Carbapenem-Resistant Enterobacteriaceae with High Rate of Autochthonous Transmission in the Arabian Peninsula. PLoS One, 2015 10(6): p. e0131372 10.1371/journal.pone.0131372 26110660 PMC4482506

[75] Boisen N., Ruiz-Perez F., Scheutz F., Krogfelt K.A., and Nataro J.P., High Prevalence of Serine Protease Autotransporter Cytotoxins among Strains of Enteroaggregative *Escherichia coli*. The American Journal of Tropical Medicine and Hygiene, 2009 80(2): p. 294–301. 19190229 PMC2660206

[76] Chandra M., Cheng P., Rondeau G., Porwollik S., and McClelland M., A single step multiplex PCR for identification of six diarrheagenic *E*. *coli* pathotypes and *Salmonella*. Int J Med Microbiol, 2013 303(4): p. 210–6. 10.1016/j.ijmm.2013.02.013 23562277

[77] Henderson I.R., Czeczulin J., Eslava C., Noriega F., and Nataro J.P., Characterization of Pic, a Secreted Protease of *Shigella flexneri* and Enteroaggregative *Escherichia coli*. Infection and Immunity, 1999 67(11): p. 5587 10.1128/IAI.67.11.5587-5596.1999 10531204 PMC96930

[78] Guyer D.M., Radulovic S., Jones F.E., and Mobley H.L., Sat, the secreted autotransporter toxin of uropathogenic *Escherichia coli*, is a vacuolating cytotoxin for bladder and kidney epithelial cells. Infect Immun, 2002 70(8): p. 4539–46. 10.1128/iai.70.8.4539-4546.2002 12117966 PMC128167

[79] Nichols K.B., Totsika M., Moriel D.G., Lo A.W., Yang J., Wurpel D.J., et al, Molecular Characterization of the Vacuolating Autotransporter Toxin in Uropathogenic *Escherichia coli* Journal of Bacteriology, 2016 198(10): p. 1487 10.1128/JB.00791-15 26858103 PMC4859599

[80] Yaseen AL-Hayali W.R., Younis Mahdy A., and A. M. Ibrahim Zaraq, Detection of auto-transporter Sat and Vat genes among *Escherichia coli* strains isolated from urinary tract infections. Tikrit Journal of Pure Science, 2019 23(10): p. 40–45.

[81] Chapman T.A., Wu X.Y., Barchia I., Bettelheim K.A., Driesen S., Trott D., et al, Comparison of virulence gene profiles of *Escherichia coli* strains isolated from healthy and diarrheic swine. Appl Environ Microbiol, 2006 72(7): p. 4782–95. 10.1128/AEM.02885-05 16820472 PMC1489375

[82] Johnson J.R. and Russo T.A., Molecular epidemiology of extraintestinal pathogenic (uropathogenic) *Escherichia coli*. Int J Med Microbiol, 2005 295(6–7): p. 383–404. 10.1016/j.ijmm.2005.07.005 16238015

[83] Schubert S., Picard B., Gouriou S., Heesemann J., and Denamur E., Yersinia high-pathogenicity island contributes to virulence in *Escherichia coli* causing extraintestinal infections. Infection and immunity, 2002 70(9): p. 5335–5337. 10.1128/iai.70.9.5335-5337.2002 12183596 PMC128248

[84] Watts R.E., Totsika M., Challinor V.L., Mabbett A.N., Ulett G.C., De Voss J.J., et al, Contribution of siderophore systems to growth and urinary tract colonization of asymptomatic bacteriuria *Escherichia coli*. Infection and immunity, 2012 80(1): p. 333–344. 10.1128/IAI.05594-11 21930757 PMC3255690

[85] Yang L., Li W., Jiang G.-Z., Zhang W.-H., Ding H.-Z., Liu Y.-H., et al, Characterization of a P1-like bacteriophage carrying CTX-M-27 in Salmonella spp. resistant to third generation cephalosporins isolated from pork in China. Scientific reports, 2017 7: p. 40710–40710. 10.1038/srep40710 28098241 PMC5241659

[86] Kleinheinz K.A., Joensen K.G., and Larsen M.V., Applying the ResFinder and VirulenceFinder web-services for easy identification of acquired antibiotic resistance and *E*. *coli* virulence genes in bacteriophage and prophage nucleotide sequences. Bacteriophage, 2014 4(2): p. e27943. 24575358 10.4161/bact.27943 PMC3926868

[87] Starikova E.V., Prianichnikov N.A., Zdobnov E., and Govorun V.M., Bioinformatics analysis of antimicrobial resistance genes and prophages colocalized in human gut metagenomes. Biomed Khim 2017 63(6): p. 508–512. 10.18097/PBMC20176306508 29251611

[88] Beyrouthy R., Robin F., Delmas J., Gibold L., Dalmasso G., Dabboussi F., et al, IS1R-mediated plasticity of IncL/M plasmids leads to the insertion of *bla* OXA-48 into the *Escherichia coli* Chromosome. Antimicrob Agents Chemother, 2014 58(7): p. 3785–90. 10.1128/AAC.02669-14 24752261 PMC4068556

[89] Beyrouthy R., Robin A. F Fau—Cougnoux G. Cougnoux A Fau—Dalmasso A. Dalmasso G Fau—Darfeuille-Michaud H. Darfeuille-Michaud A Fau—Mallat F. Mallat H Fau—Dabboussi, et al, Chromosome-mediated OXA-48 carbapenemase in highly virulent *Escherichia coli*. Journal of Antimicrobial Chemotherapy, 2013 68(1460–2091 (Electronic)): p. 1558–1561. 10.1093/jac/dkt051 23447140

[90] Turton J.F., Doumith M., Hopkins K.L., Perry C., Meunier D., and Woodford N., Clonal expansion of *Escherichia coli* ST38 carrying a chromosomally integrated OXA-48 carbapenemase gene. J Med Microbiol, 2016 65(6): p. 538–46. 10.1099/jmm.0.000248 26982715

