# Supplementary figures and images for: Clinical and molecular characteristics of carbapenem non-susceptible Escherichia coli: A nationwide survey from Oman
Source: PLoS One. 2020 Oct 9;15(10):e0239924. doi: 10.1371/journal.pone.0239924 (PMC7546912; doi:10.1371/journal.pone.0239924)

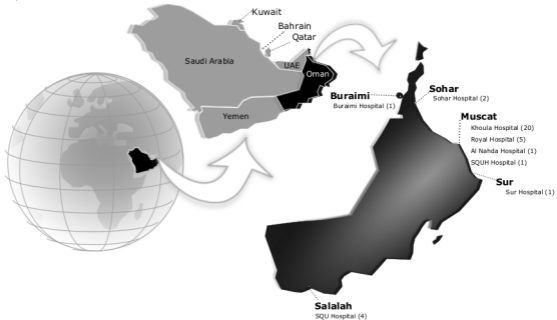

Supplement: S1 Fig — The 35 samples were obtained from 8 tertiary hospitals in Oman. Four of them are located in the capital, Muscat. Buraimi Hospital is located at the border with United Arab Emirates (UAE) whereas Sultan Qaboos Hospital (SQH) in Salalah is located near Yemen. Names of the cities in Oman’s map represented with bold font whereas hospitals’ names were listed below them. The number of samples obtained from each hospitals was represented within parenthesis. (PDF) [file pone.0239924.s001.pdf]
