# Supplementary material for: Clinical and molecular characteristics of carbapenem non-susceptible Escherichia coli: A nationwide survey from Oman
Source: PLoS One. 2020 Oct 9;15(10):e0239924. doi: 10.1371/journal.pone.0239924 (PMC7546912; doi:10.1371/journal.pone.0239924)
Supplement: S2 Fig — Heatmap shows phenotypic readings, ARGs in antibiotic classes other than β-lactam, plasmid replicons and prophages for the studied 35 strains. (PDF) [file pone.0239924.s002.pdf]

Phenotypic tests*

Strains (ID_ST_Phylogroup)	β-lactam classification	Disk Diffusion (mm): Imipenem (IMP)	Meropenem (MEM)	Ertapenem (ERT)	Cefotaxime (CTX)	Ceftazidime (CAZ)	Ciprofloxacin (CIP)	Ceftazidime-avibactam (CAZ-AV)	Broth Microdilution (mg/L): Colistin	Tigecycline (TGC)	Agar Dilution (mg/L): Fosfomycin	ROSCO DIAGNOSTICA	Carba NP
**OM898**_ST1702_A	NDM+ESBL+AmpC	12	11	6	6	6	6	NA	<1	<0.5	<1	MBL	NDe
**OM852**_ST1702_A	NDM+ESBL+AmpC	8	6	6	6	6	6	NA	<1	<0.5	<1	MBL	NDe
**OM1071**_ST167_A	OXA-48-like+ESBL+AmpC	24	23	15	6	6	6	15	<1	<0.5	<1	OXA-48/ESBL	NDe
**OM1626**_ST167_A	NDM+ESBL	12	6	6	6	6	6	NA	<1	<0.5	<1	MBL	NDe
**OM855**_ST167_A	NDM+ESBL	12	6	6	6	6	6	NA	<1	<0.5	<1	MBL	NDe
**OM979**_ST617_A	NDM+ESBL	19	18	14	6	6	6	NA	<1	<0.5	<1	ND	NDe
**OM5639**_ST617_A	AmpC+ESBL	24	26	22	6	6	6	NA	<1	<0.5	<1	OXA-48/ESBL	Negative
**OM693**_ST617_A	ESBL	29	29	24	6	6	6	NA	<1	<0.5	<1	OXA-48/ESBL	NDe
**OM1398**_ST540_A	NDM+ESBL+AmpC	14	13	8	6	6	6	NA	<1	<0.5	<1	MBL	NDe
**OM211**_ST361_A	ESBL	24	22	12	6	6	6	NA	<1	<0.5	<1	OXA-48/ESBL	Negative
**OM112**_ST361_A	AmpC	24	24	28	6	6	6	NA	<1	<0.5	<1	ESBL+PORIN LOSS	Negative
**OM1576**_ST46_A	NDM+ESBL	16	14	11	6	6	6	NA	<1	<0.5	<1	MBL	NDe
**OM147**_ST101_B1	NDM+ESBL	14	13	6	6	6	6	NA	<1	<0.5	<1	MBL	NDe
**OM79**_ST101_B1	NDM+ESBL	12	10	6	6	6	6	NA	<1	<0.5	<1	MBL	NDe
**OM1341**_ST156_B1	NDM+OXA-48-like+ESBL	18	16	7	6	6	6	NA	<1	<0.5	<1	OXA-48/ESBL	NDe
**OM664**_ST448_B1	NDM+ESBL	11	15	10	6	6	6	NA	<1	<0.5	<1	MBL	NDe
**OM561**_ST410_C	NDM+ESBL	14	15	12	6	6	6	NA	<1	<0.5	<1	MBL	NDe
**OM1273**_ST410_C	OXA-48-like+ESBL+AmpC	22	22	16	6	6	6	18	<1	<0.5	<1	OXA-48/ESBL	NDe
**OM1301**_ST410_C	NDM+OXA-48-like+AmpC+ESBL	10	8	6	6	6	6	NA	<1	<0.5	<1	ND	NDe
**OM853**_ST652_C	ESBL	30	32	32	6	13	14	NA	<1	<0.5	<1	ESBL+PORIN LOSS	NDe
**OM1609**_ST6870_F	AmpC	29	29	29	29	27	30	NA	<1	<0.5	<1	ESBL+PORIN LOSS	NDe
**OM78**_ST1340_F	ESBL	28	30	27	6	11	6	NA	<1	<0.5	<1	ESBL+PORIN LOSS	NDe
**OM260**_ST131_B2	AmpC+ESBL	28	32	31	6	13	6	NA	<1	<0.5	<1	ESBL+PORIN LOSS	NDe
**OM333**_ST73_B2	none	29	30	34	27	25	34	NA	<1	<0.5	<1	ESBL+PORIN LOSS	NDe
**OM1692**_ST127_B2	ESBL	29	29	29	8	21	31	NA	<1	<0.5	<1	ESBL+PORIN LOSS	NDe
**OM1136**_ST1193_B2	NDM+ESBL+AmpC	19	19	17	6	6	6	NA	<1	<0.5	<1	MBL	NDe
**OM126**_ST2914_D	OXA-48-like+ESBL	20	21	17	29	24	25	26	<1	<0.5	<1	MBL	NDe
**OM1168**_ST405_D	NDM+ESBL	12	8	6	6	6	6	NA	<1	<0.5	<1	MBL	NDe
**OM82**_ST405_D	ESBL	29	30	22	6	6	6	NA	<1	<0.5	<1	ESBL+PORIN LOSS	NDe
**OM839**_ST405_D	ESBL	25	29	21	6	6	6	NA	<1	<0.5	<1	ESBL+PORIN LOSS	NDe
**OM150**_ST2659_D	ESBL	30	30	24	6	6	6	NA	<1	<0.5	<1	ESBL+PORIN LOSS	NDe
**OM481**_ST38_D	OXA-48-like	20	21	15	17	25	24	26	<1	<0.5	<1	OXA-48/ESBL	NDe
**OM1433**_ST38_D	OXA-48-like+ESBL	23	22	16	6	23	28	28	<1	<0.5	<1	OXA-48/ESBL	NDe
**OM234**_ST38_D	OXA-48-like+ESBL	19	19	15	6	10	25	25	<1	<0.5	<1	OXA-48/ESBL	NDe
**OM347**_ST38_D	OXA-48-like+ESBL+AmpC	21	21	13	6	6	26	18	<1	<0.5	<1	OXA-48/ESBL	NDe

Resistance genes for antibiotic classes other than β-lactam**

Strains	Aminoglycosides – AACs: *aac*(3)-IId	*aac*(3)-IIa	*aac*(6')-Ib	*aac*(6')Ib-cr	ANTs: *aad*A2	*aad*A1	*aad*A5	*aad*A12	*aad*A4	APHs: *aph*(3'')-VIa	*aph*(3')-Ia	16S modifying enzymes: *rmt*B	*rmt*C	streptomycin resistance genes: *str*B	*str*A	Fluoroquinolones: *aac*(6')Ib-cr	*qnr*S1	*qnr*B4	Tetracycline: *tet*(A)	*tet*(B)	*tet*(D)	folate synthesis inhibitors: *sul1*	*sul2*	*dfr*A12	*dfr*A17	Phenicol: *cat*B3	Total
OM898_ST1702_A				■			■							■	■	■	■		■			■	■		■	■	11
OM852_ST1702_A				■			■							■	■	■	■		■			■	■		■	■	11
OM1071_ST167_A				■			■					■				■	■		■			■			■	■	9
OM1626_ST167_A				■	■							■				■			■			■		■		■	8
OM855_ST167_A				■	■							■				■			■			■		■		■	8
OM979_ST617_A				■	■		■							■	■	■				■		■	■	■	■	■	12
OM5639_ST617_A				■			■									■				■		■			■	■	7
OM693_ST617_A				■	■									■	■	■						■	■	■		■	9
OM1398_ST540_A					■							■										■		■			4
OM211_ST361_A							■													■		■			■		4
OM112_ST361_A																											0
OM1576_ST46_A				■	■							■				■			■			■		■		■	8
OM147_ST101_B1					■							■								■		■		■			5
OM79_ST101_B1			■		■	■						■								■		■	■	■			8
OM1341_ST156_B1					■				■												■	■		■			5
OM664_ST448_B1				■			■							■	■	■				■		■	■		■		9
OM561_ST410_C				■			■									■			■			■			■	■	7
OM1273_ST410_C				■			■							■	■	■	■			■		■	■		■	■	11
OM1301_ST410_C				■	■		■					■		■	■	■				■		■	■	■	■	■	13
OM853_ST652_C																				■							1
OM1609_ST6870_F				■			■									■				■		■			■		6
OM78_ST1340_F											■																1
OM260_ST131_B2				■			■									■		■	■			■			■	■	8
OM333_ST73_B2																											0
OM1692_ST127_B2																				■							1
OM1136_ST1193_B2	■			■						■			■	■	■	■				■		■	■		■	■	12
OM126_ST2914_D								■						■	■		■		■				■				6
OM1168_ST405_D					■							■		■	■					■		■	■	■			8
OM82_ST405_D		■		■			■									■				■		■			■	■	8
OM839_ST405_D				■			■									■				■		■			■	■	7
OM150_ST2659_D				■			■							■	■	■						■	■		■	■	9
OM481_ST38_D							■														■	■					3
OM1433_ST38_D							■															■			■		3
OM234_ST38_D			■			■											■					■					4
OM347_ST38_D																	■										1
	1	1	2	19	11	2	17	1	1	1	1	9	1	11	11	19	7	1	9	15	2	28	12	11	17	17	227

Plasmid Replicon types***

Strains	IncF: II	IA	IB	IncX: 1	3	4	IncI1	IncA/C2	IncL/M	IncN	IncY	IncR	IncQ1	Col: BS512	KP3	pVC	156	p0111	Total
OM898_ST1702_A	2	4					C												3
OM852_ST1702_A	2	4					C												3
OM1071_ST167_A	33	4			■		B			F					■				6
OM1626_ST167_A	36	4												■					3
OM855_ST167_A	36	4												■					3
OM979_ST617_A	31	4	1											■					4
OM5639_ST617_A	31	4	1		■		A							■					6
OM693_ST617_A	1	2	33																3
OM1398_ST540_A	2																		1
OM211_ST361_A	36		32	■							■		■						5
OM112_ST361_A	1		49				B												3
OM1576_ST46_A	1	1	20											■					4
OM147_ST101_B1	22	1	20													■			4
OM79_ST101_B1	2	1	20		■							■				■			6
OM1341_ST156_B1									■										1
OM664_ST448_B1	48	1	49		■								■						5
OM561_ST410_C	31	4	1		■				■							■			6
OM1273_ST410_C	1	1	49		■								■		■				6
OM1301_ST410_C	1	1	49		■						■		■	■	■				8
OM853_ST652_C	31	4	1											■			■		5
OM1609_ST6870_F	1	1	1				A												4
OM78_ST1340_F	18		1																2
OM260_ST131_B2	36	4	1				D			5									5
OM333_ST73_B2				■															1
OM1692_ST127_B2	29		10				57										■		4
OM1136_ST1193_B2		1	10					■					■	■			■		6
OM126_ST2914_D	2		53						■										3
OM1168_ST405_D	1	1			■	■				6				■				■	7
OM82_ST405_D	36	4	10																3
OM839_ST405_D	1	1	49				A									■			5
OM150_ST2659_D	36	4	1				E												4
OM481_ST38_D	24		1																2
OM1433_ST38_D																			0
OM234_ST38_D	16					■													2
OM347_ST38_D					■	■	C												3
	30	23	23	2	9	3	11	1	3	3	2	1	5	9	3	4	3	1	136

Prophages

Strains	Intact (score >90)	Questionable (score 70-90)	Incomplete (score < 70)	Total
OM898_ST1702_A	2		1	3
OM852_ST1702_A	2		1	3
OM1071_ST167_A	2		1	3
OM1626_ST167_A	2		3	5
OM855_ST167_A	2		3	5
OM979_ST617_A	2	1		3
OM5639_ST617_A	4	1	2	7
OM693_ST617_A	4		1	5
OM1398_ST540_A	1	1	4	6
OM211_ST361_A	2	1	4	7
OM112_ST361_A	2	1	1	4
OM1576_ST46_A		1	2	3
OM147_ST101_B1	5	1	3	9
OM79_ST101_B1	4	2	1	7
OM1341_ST156_B1	1		3	4
OM664_ST448_B1	3		3	6
OM561_ST410_C	1		3	4
OM1273_ST410_C		1	4	5
OM1301_ST410_C	1		4	5
OM853_ST652_C	1	2	3	6
OM1609_ST6870_F	3	1	3	7
OM78_ST1340_F		1	1	2
OM260_ST131_B2	4	2	3	9
OM333_ST73_B2	3	1	3	7
OM1692_ST127_B2	4	1	2	7
OM1136_ST1193_B2	3	2	5	10
OM126_ST2914_D	3			3
OM1168_ST405_D	3	1	2	6
OM82_ST405_D	1		2	3
OM839_ST405_D	3	1	5	9
OM150_ST2659_D	2		6	8
OM481_ST38_D	5	1	1	7
OM1433_ST38_D	1	1	5	7
OM234_ST38_D	2		4	6
OM347_ST38_D	2		7	9
	80	24	96	200

*Meropenem reading > 28 mm were shaded as the cut-off value for carbapenemase screening according to EUCAST guidelines. Only isolates producing OXA-48-like carbapenemase were screened for ceftazidime-avibactam as NA: non-applicable.
In ROSCO DIAGNOSTICA, readings inconsistant with genomic data were shaded, "NDe" not determined due to equivocal readings. Carba NP test was performed only for isolates with no carbapenmase genes but cut-off values <28 mm, "NDo" not done.
**No resistance genes were detected for colistin, fosfomycin and tigecycline. Besides, phenotypic tests showed all isolates were susceptible to these antibiotics.
***Variants of plasmid MLST subtyping for IncF, IncI1 and IncN replicons are presented with white characters in the corresponding cells. For IncI1 and IncN, digits indicate known STs assigned by MLSTplasmid web tool at CGE database while letters were assigned arbitrarly for unkown STs as for IncN [*kor*A, *rep*N,*tra*J]; F [0:1:0] while for IncI1 [*ard*A:*trb*A:*pil*L:*sog*S:*rep*I1]; A [5:15:0:0:4]; B [5:15:3:0:4]; C [2:15:0:0:4]; D [2:16:7:1:1]; E [2:3:5:7:5].
