# Supplementary material for: Clinical and molecular characteristics of carbapenem non-susceptible Escherichia coli: A nationwide survey from Oman
Source: PLoS One. 2020 Oct 9;15(10):e0239924. doi: 10.1371/journal.pone.0239924 (PMC7546912; doi:10.1371/journal.pone.0239924)
Supplement: S1 Table — (PDF) [file pone.0239924.s003.pdf]

**S1 Table: Genomic quality of sequenced strains and their corresponding SRA accession numbers.**

| ID | | RAW READS INFORMATION | | | | | | | | ASSEMBLY INFORMATION | | | | | |
|---|---|---|---|---|---|---|---|---|---|---|---|---|---|---|---|
| Sample ID | SRA accession | Total Reads | Median Insert Size | Duplication Rate | Mapped Rate | Coverage 10X | Coverage 30X | Coverage 50X | Coverage 100X | N50 | N90 | Length | Contigs Number | Gaps Number | N-count |
| OM1071 | SAMN11840205 | 4,534,780 | 164 | 3.6% | 77.7% | 99.5% | 87.3% | 62.4% | 24.0% | 119199 | 18374 | 5096380 | 263 | 1 | 10 |
| OM112 | SAMN11840223 | 2,833,451 | 590 | 2.8% | 85.2% | 98.8% | 72.6% | 41.8% | 10.5% | 120711 | 32683 | 4946148 | 106 | 5 | 889 |
| OM1136 | SAMN11840193 | 5,058,853 | 492 | 3.3% | 76.6% | 99.5% | 98.8% | 96.1% | 42.1% | 232875 | 37384 | 5152949 | 112 | 8 | 609 |
| OM1168 | SAMN11840201 | 3,807,172 | 533 | 3.6% | 67.4% | 98.9% | 70.2% | 42.3% | 15.8% | 123272 | 17438 | 5532892 | 318 | 8 | 71 |
| OM126 | SAMN11840207 | 7,794,819 | 155 | 7.5% | 67.4% | 99.4% | 85.1% | 60.4% | 33.1% | 199949 | 43089 | 4986467 | 146 | 1 | 10 |
| OM1273 | SAMN11840206 | 5,062,054 | 613 | 3.3% | 87.4% | 99.3% | 93.7% | 82.3% | 48.4% | 218208 | 45821 | 4768338 | 94 | 11 | 976 |
| OM1301 | SAMN11840210 | 2,596,955 | 603 | 2.5% | 77.4% | 95.0% | 59.6% | 35.8% | 8.7% | 154021 | 38418 | 4984107 | 102 | 15 | 302 |
| OM1341 | SAMN11840211 | 5,061,749 | 164 | 4.5% | 76.6% | 98.8% | 79.4% | 58.9% | 30.6% | 144515 | 29634 | 5031736 | 224 | 6 | 60 |
| OM1398 | SAMN11840192 | 10,601,814 | 126 | 9.9% | 79.5% | 99.8% | 99.0% | 93.9% | 60.9% | 123306 | 26668 | 4906395 | 180 | 0 | 0 |
| OM1433 | SAMN11840208 | 2,559,405 | 627 | 2.7% | 81.7% | 98.5% | 78.6% | 44.7% | 2.6% | 154613 | 29443 | 5014579 | 158 | 10 | 1018 |
| OM147 | SAMN11840195 | 5,779,777 | 178 | 4.6% | 79.6% | 99.4% | 87.0% | 70.0% | 41.6% | 150780 | 38476 | 4899772 | 97 | 2 | 20 |
| OM150 | SAMN11840217 | 8,447,972 | 163 | 6.6% | 69.1% | 99.6% | 97.3% | 84.6% | 49.6% | 103390 | 20258 | 5249550 | 279 | 2 | 264 |
| OM1576 | SAMN11840200 | 7,643,686 | 151 | 5.5% | 78.1% | 99.6% | 93.8% | 82.0% | 54.8% | 113293 | 20491 | 4710482 | 202 | 3 | 30 |
| OM1609 | SAMN11840213 | 4,698,763 | 144 | 10.6% | 70.3% | 99.5% | 98.9% | 92.2% | 41.1% | 197135 | 33125 | 5024084 | 109 | 7 | 192 |
| OM1626 | SAMN11840202 | 6,194,175 | 192 | 4.2% | 84.4% | 99.9% | 99.1% | 92.8% | 56.8% | 137180 | 34718 | 4940041 | 175 | 7 | 256 |
| OM1692 | SAMN11840222 | 6,142,330 | 188 | 4.1% | 69.3% | 99.4% | 98.5% | 91.7% | 41.6% | 278373 | 30769 | 5285160 | 115 | 4 | 346 |
| OM211 | SAMN11840218 | 5,733,111 | 175 | 4.3% | 81.9% | 99.8% | 97.8% | 86.1% | 40.9% | 92988 | 30036 | 5019162 | 139 | 0 | 0 |
| OM234 | SAMN11840203 | 5,499,127 | 182 | 3.9% | 81.4% | 99.5% | 94.0% | 83.5% | 47.2% | 165731 | 34459 | 5076719 | 163 | 6 | 298 |
| OM260 | SAMN11840212 | 6,761,043 | 167 | 4.5% | 65.9% | 99.3% | 97.0% | 83.3% | 41.3% | 196019 | 21478 | 5389896 | 237 | 2 | 211 |
| OM333 | SAMN11840224 | 5,357,255 | 180 | 4.4% | 76.4% | 99.0% | 87.7% | 71.7% | 39.0% | 202615 | 28541 | 5127360 | 110 | 2 | 20 |
| OM347 | SAMN11840204 | 5,401,215 | 192 | 4.3% | 78.6% | 99.3% | 89.6% | 73.1% | 38.5% | 209442 | 40618 | 5035611 | 102 | 5 | 50 |
| OM481 | SAMN11840209 | 3,633,167 | 625 | 3.7% | 77.9% | 94.9% | 73.4% | 54.4% | 22.6% | 172109 | 50040 | 5064280 | 105 | 16 | 1083 |
| OM561 | SAMN11840196 | 2,551,897 | 654 | 2.1% | 81.2% | 98.2% | 81.7% | 45.1% | 0.7% | 131825 | 41339 | 4926496 | 86 | 6 | 400 |
| OM5639 | SAMN11840214 | 3,391,250 | 590 | 2.3% | 81.5% | 98.4% | 86.2% | 65.1% | 12.6% | 97083 | 33122 | 4855636 | 168 | 5 | 260 |
| OM664 | SAMN11840197 | 3,986,832 | 573 | 3.4% | 83.4% | 95.7% | 76.4% | 59.9% | 30.6% | 153834 | 38574 | 4804883 | 101 | 17 | 520 |
| OM693 | SAMN11840219 | 6,937,699 | 157 | 4.7% | 77.6% | 99.8% | 97.9% | 89.4% | 53.4% | 120148 | 25815 | 4969452 | 181 | 5 | 284 |
| OM78 | SAMN11840215 | 5,303,939 | 198 | 3.8% | 81.8% | 99.5% | 95.7% | 86.4% | 48.9% | 150891 | 44078 | 4956714 | 87 | 2 | 300 |
| OM79 | SAMN11840194 | 4,427,065 | 220 | 3.6% | 73.3% | 98.9% | 80.5% | 58.0% | 25.7% | 132104 | 31960 | 4892695 | 178 | 1 | 10 |
| OM82 | SAMN11840216 | 5,525,508 | 173 | 4.8% | 75.4% | 98.8% | 82.3% | 63.6% | 34.7% | 108993 | 27399 | 4966530 | 138 | 5 | 50 |
| OM839 | SAMN11840220 | 5,485,109 | 180 | 10.0% | 71.6% | 99.5% | 99.0% | 98.3% | 71.2% | 99264 | 19429 | 5391232 | 214 | 0 | 0 |
| OM852 | SAMN11840190 | 4,422,103 | 274 | 13.0% | 72.1% | 99.7% | 98.2% | 85.6% | 38.3% | 136972 | 28671 | 5094421 | 194 | 0 | 0 |
| OM853 | SAMN11840221 | 3,384,266 | 603 | 3.4% | 77.2% | 98.0% | 72.1% | 47.6% | 16.2% | 144204 | 28659 | 4957600 | 193 | 6 | 507 |
| OM855 | SAMN11840198 | 4,173,897 | 504 | 3.4% | 79.6% | 99.4% | 77.6% | 53.1% | 24.1% | 163050 | 34792 | 4930022 | 131 | 6 | 418 |
| OM898 | SAMN11840191 | 5,680,129 | 196 | 4.1% | 77.8% | 99.8% | 93.3% | 75.2% | 37.9% | 127191 | 24425 | 5038522 | 154 | 0 | 0 |
| OM979 | SAMN11840199 | 3,531,528 | 541 | 2.8% | 79.5% | 98.3% | 80.1% | 58.1% | 18.9% | 126253 | 19604 | 4935146 | 290 | 14 | 408 |
