# Supplementary material for: Clinical and molecular characteristics of carbapenem non-susceptible Escherichia coli: A nationwide survey from Oman
Source: PLoS One. 2020 Oct 9;15(10):e0239924. doi: 10.1371/journal.pone.0239924 (PMC7546912; doi:10.1371/journal.pone.0239924)
Supplement: S2 Table — (PDF) [file pone.0239924.s004.pdf]

S2 Table: Demogaphic data.

ID	Hosp.	Dep.	Nationality	Age	Sex	Specimen	Isolation Month	Outcome	Underlying Disease	Antibiotic Usage	Travel History
OM78	Nahda	Med.	Omani	81	F	Blood	Jan	Died	DM, HTN, bed sores, arthritis	AMI, MER	NA
OM79	Khoula	Ped.	Omani	0	F	Fecal screening	Jan	Improved	Gangrene	CIP	India
OM82	Royal	Surg.	Omani	62	M	Swab	Jan	Died	Upper gastrointestinal bleeding	None	India
OM112	Royal	Med.	Omani	70	F	Wound	Jan	Improved	HTN, DM, CAD	PTZ, CRO, CLR	NA
OM126	Khoula	Plast. Surg.	Omani	2	M	Fecal screening	Jan	Improved	Burns, G6PD	CXM, CLX	NA
OM147	Khoula	Ped.	Omani	0	F	Urine	Feb	Improved	Vascular malformation	CIP	India
OM150	Royal	ICU	Omani	59	M	Sputum	Feb	Died	DM, ESRD	MER, CLR	NA
OM211	Royal	Med.	Indian	48	M	Fecal screening	Feb	Improved	HIV	PTZ, SXT, PEN	NA
OM234	Khoula	Surg.	Omani	54	M	Fecal screening	Feb	Improved	Colostomy status, perineal wound, DM, HTN, fornier gangrene necrotizing fasciitis, postoperative infectior	CIP, MTZ, MER, CLX, PTZ	India
OM260	Khoula	Surg.	Indian	57	F	Biopsy	Feb	Improved	DM, HTN, renal failure, asthma	PTZ, AML, MTZ	India
OM333	Buraimi	RDU	Omani	80	M	Blood	Mar	Died	DM, HTN	MER	NA
OM347	Khoula	Neur. Surg.	Omani	55	F	Wound	Mar	Referred to other hosp.	Spine fracture, morbid obesity, bed sores HTN , DM	MTZ, AMI, COL, MER, PTZ, VAN	India
OM481	Khoula	Surg.	Yemeni	7	M	Fecal screening	Mar	Improved	Armed conflict in Yemen, liver damage	CXM	NA
OM561	Khoula	OPC	Omani	58	M	Urine	Apr	Improved	HTN, DM, CABG, indwelling urinary catheter	AMX	India
OM664	Khoula	Orthop.	Omani	47	M	Fecal screening	Apr	Improved	Lower back surgery (decompression)	NA	India
OM693	Royal	Nephrol.	Omani	55	M	Fecal screening	Apr	Died	HTN, DM, kidney transplant, graft dysfunction	MER, STX	NA
OM839	Khoula	OPC	Omani	74	F	Urine	May	Improved	DM, HTN, hyperlipidemia, renal cyst, lumbar spondylitis, spondylolistheis, osteoporosis	NIT	India
OM852	Khoula	Surg.	Yemeni	50	M	Wound	May	Improved	Armed conflict in Yemen	MTZ, PTZ, COL, VAN, CXM	NA
OM853	Khoula	ICU	Yemeni	20	M	Fecal screening	May	Improved	Armed conflict in Yemen, cervical spine	CFZ	NA
OM855	Khoula	Orthop.	Yemeni	23	M	Fecal screening	May	Improved	Armed conflict in Yemen, paraplegic since one month	CFZ	NA
OM898	Khoula	ICU	Yemeni	36	M	Biopsy	Jun	Improved	Armed conflict in Yemen, Spinal injury, paraplegia	CRO, VAN, MER, COL, PTZ, CIP	NA
OM979	Khoula	Surg.	Yemeni	19	F	Fecal screening	Jun	Improved	Armed conflict in Yemen abdominal blast injury	PTZ, MTZ, CXM	NA
OM1071	Khoula	Surg.	Omani	68	M	Drain fluid	Jun	Improved	Colorectal cancer, laparotomy done 12 days earlier	PTZ, MTZ	NA
OM1136	Sohar	Med.	Omani	66	M	Tracheal isolate	Jul	Improved	Tracheostomy status, bedridden	CRO, PTZ	NA
OM1168	SQH	Surg.	Yemeni	8	F	Fecal screening	Jul	Improved	Armed conflict in Yemen, femur fracture, gas gangrene	CAZ	NA
OM1273	SQH	Surg.	Yemeni	40	M	Wound	Aug	Improved	Armed conflict in Yemen, gun shot injury abdomen	PTZ, AMI, CTX, CRO, CIP, MER	NA
OM1301	Sohar	OPC	Omani	82	M	Urine	Aug	Improved	Prostate hyperplasia, hyperthyroidism, atrial fibrillation	NA	NA
OM1341	Khoula	Orthop.	Omani	71	M	Fecal screening	Aug	Improved	Rib fracture	CXM, MTZ	NA
OM1398	SQH	Surg.	Omani	59	F	Fecal screening	Aug	Improved	DM, HTN	MER, PTZ, SXT, CIP	NA
OM1433	Khoula	Ped.	Omani	0	M	Umbilical isolate	Sep	Improved	Preterm birth	None	NA
OM1576	Khoula	Neur. Surg.	Omani	29	M	Wound	Sep	Referred to other hosp.	Operation for skull defect, bilateral bed sores.	CXM, PTZ, MER, COL	India
OM1609	Sohar	Surg.	Omani	83	M	Wound	Sep	Improved	Bedridden, Decubitus ulcer, HTN, DM and old CVA	CIP	NA
OM1626	SQH	Cardio surg.	Omani	48	M	Wound	Sep	Improved	DM, HTN, CAD, CABG	DOX, CIP	no
OM1692	Khoula	neonatal ICU	Omani	1	M	Urine	Sep	Hospitalised	Multiple congenital anomalies, frequent colonization of urinary and respiratory tract with *Klebsiella*	AML, PTZ, SXT, VAN, CXM, CTX	NA
OM5639	SQUH	Orthop.	Yemeni	20	M	Biopsy	Oct	Hospitalised	Armed conflict in Yemen	NA	NA

Antibiotic Abbreviations: AMI-amikacin, AML- amoxicillin-clavulanic acid, AMX-amoxicillin, CAZ-ceftazidime, CFZ-cefazoline, CIP-ciprofloxacin, CLR-clarithromycin, COL-colistin, CRO-ceftriaxone, CTX-cefotaxime, CLX-cloxacillin, CXM-cefuroxime, DOX-doxycycline, NIT-nitrofurantoin, MER-meropenem, MTZ-metronidazole, PEN- benzylpenicillin, , VAN-vancomycin, PTZ-piperacillin-tazobactam, SXT-trimethoprim/sulfamethoxazole.

Other abbreviations: CAD-coronary artery disease, CABG-coronary artery bypass graft, CVA-cerebrovascular accident, DM-diabetes mellitus, ESRD-end stage renal disease, G6PD-glucose-6-phosphate dehydrogenase deficiency, HIV-human immunodeficiency virus, HTN-hypertension, ICU-intensive care unit, IP-Inpatient, NA-not available, OPC-out patient clinic, RDU-renal dialysis unit.
