# Supplementary material for: Clinical and molecular characteristics of carbapenem non-susceptible Escherichia coli: A nationwide survey from Oman
Source: PLoS One. 2020 Oct 9;15(10):e0239924. doi: 10.1371/journal.pone.0239924 (PMC7546912; doi:10.1371/journal.pone.0239924)
Supplement: S3 Table — (PDF) [file pone.0239924.s005.pdf]

S3 Table: Genetic alterations in regulatory proteins for porins and AcrAB-TolC efflux pump.

ID	Porin (influx) regulators			AcrAB-TolC efflux pump regulators						
	**OmpC**	**OmpF**	**OmpR**	**AcrR**	**MarA**	**MarB**	**MarR**	**SoxS**	**SoxR**	**EnvZ**
OM78	V24I	G48D		**V29G**		H44Q				
	K48E	E51V								
	K48_D49delinsNS	M60K								
	V50K									
	N235T									
OM79		N31fs		**T5N**			**G103S**			
		S201T					**Y137H**			
		E203D								
		G211A								
		I295L								
OM82	V15A	G48D		**V29G**			**K62R**			
	A18T	E51V		T213I						
	N20H	M60K		N214T						
	V24I	T99K								
	L32V	Y112F								
	V38A	V115A								
	D39V	F118I								
		R185_D186insA								
		D186G								
		T187I								
		A188P								
		R189E								
		Y204F								
		N220D								
		L221A								
		Q225E								
		P226F								
		L227R								
		N229H								
		N229K								
		Y253fs								
OM126						S5L	**Y137H**		**T38S**	A25V
						A17T			**G74R**	T466A
						V20I				
						H44Q				
OM147	S7A	N31fs		**T5N**		H44Q				
	V15A									
	N20H									
	V24I									
OM150	V24I	G48D R42L	R42L			S5L				R42L
	K48S	E51V				L12F				
	V319F	M60K				A17T				
		N74T				V20I				
		S75A				H44Q				
		D76H								
		T99K								
		Y112F								
		V115A								
		F118I								
		D186G								
		T187I								
		A188P								
		R189E								
		S201T								
		E203N								
		Y204F								
		V210G								
		G211A								
		N220D								
		L221A								
		Q225E								
		P226F								
		L227R								
		N229L								
		D278N								
		V279F								

ID	Porin (influx) regulators			AcrAB-TolC efflux pump regulators						
	OmpC	OmpF	OmpR	AcrR	MarA	MarB	MarR	SoxS	SoxR	EnvZ
		N315K								
		F317V								
		E318D								
		I336K								
OM211	V15A	V33L		**V29fs**						
	N20H	I336N								
	V24I									
	L32V									
	VAL38A									
	L296V									
OM234	A18T					H44Q	**G103S**		**T38S**	A25V
	N20H						**Y137H**		**G74R**	T466A
	V24I									I86L
	L32V									
	V38A									
	D39V									
	D46N									
	D46_N47delinsED									
	T115S									
	N165D									
	G216A									
	I218V									
	A226D									
	T229N									
	A230T									
	V319A									
	D344N									
	N345D									
OM260	V15A	N315K								
	N20H	F317G								
	V24I	E318D								
	L32V	I336K								
	V38A									
	D39V									
	K48S									
	D49S									
	V50delinsAK									
	V50E									
	F149V									
	T155S									
	D156N									
	G216A									
	I218V									
	A284V									
	L296V									
	A297G									
	L299V									
	Q300Y									
	R308fs									
	L317N									
	V319F									
	D320E									
	Q346R									
	N357D									
OM333	V15A	S164N								
	A18T	N165D								
	N20H	V177A								
	V24I	L180Q								
	L32V	A224fs								
	D320E	Q225L								
	K338I	G228fs								
		N229K								
		T276A								
		D278N								
		V279fs								
		L280fs								
		I295V								
		T298_K299delinsLQ								
OM347	V15A	G48D				S5L	**Y137H**		**T38S**	A25V

ID	Porin (influx) regulators			AcrAB-TolC efflux pump regulators						
	**OmpC**	**OmpF**	**OmpR**	**AcrR**	**MarA**	**MarB**	**MarR**	**SoxS**	**SoxR**	**EnvZ**
	A18T					L12F	**K62R**		**G74R**	T466A
	N20H					A17T				
	V24I					V20I				
	L32V					H44Q				
OM481		G48D							**T38S**	A25V
		E51V							**G74R**	T466A
		M60K								
		Y112F								
		V115A								
		F118I								
		R185_D186insA								
		T187I								
		A188P								
		R189E								
		Y204F								
		N220D								
		L221A								
OM561	A226D						**Y137H**			
	T229N									
	A230fs									
OM664	V24I	A63M				H44Q				
	K48E	I73V								
	K48_D49delinsNS	N74T								
	V50K	S75_D76delinsDH								
	Q54K	N86_F87delinsKI								
	S85N	S201T								
	N88S	E203N								
	E89D	V210G								
	N91S	G211A								
	G137D									
	N165D									
	D208N									
	G216A									
	I218V									
	A226D									
	T229N									
	A230T									
	A231fs									
	N235T									
	N357D									
OM693	V24I	Ile336L								
	L32V									
	V38A									
	D39V									
	L296M									
	K317N									
	V319S									
	D320E									
	V321I									
OM852									**A111T**	
OM853	V186I									
	D192G									
	A226D									
	T229S									
	A230fs									
	L296V									
	R308fs									
OM855				**V29fs**					**A111T**	
OM898		N315K		**V29fs**					**A111T**	
		F317V								
		E318D								
OM1071		A302G		**V29fs**					**A111T**	
		V305I								
		V311E								
		V314M								
		N315K								
		F317V								

ID	Porin (influx) regulators			AcrAB-TolC efflux pump regulators						
	OmpC	OmpF	OmpR	AcrR	MarA	MarB	MarR	SoxS	SoxR	EnvZ
		E318D								
		I336K								
OM1136	N47D	Q225L			S127N	S5L	**Y137H**		**G74R**	A25V
	D49S	N229K				A10T	**G103S**			T466A
	V186I					A33G	**S3N**			
	D192G					H44Q				
	G216A									
	I218V									
	N235T									
	G309_Y310insRN									
OM1168	A18T	V115A		K80fs		H44Q	**Y137H**		**T38S**	A25V
	V24I	F118I					**G103S**		**G74R**	T466A
	L32V						**K62R**			
	V38A									
	D46_N47delinsED									
	V96L									
	Q104A									
	V106A									
	F109I									
	N165D									
OM1273						H44Q	**Y137H**			
							**G103S**			
OM1341	G137D	T19A		V43fs		H44Q	**G103S**	**A12S**		
	G216A	I25V								
	I218V	V33L								
	L296V	V40D								
	G308fs	N165D								
		L180Q								
		S199fs								
		E318D								
		I336K								
OM1398	S7A	T19A		S87*		H44Q	**Y137H**			
	V15A	I25V					**G103S**			
	N20H	V33L					A70E			
	V38A	A39V								
	D39V	V40D								
	D46N	G48D								
	D46_N47delinsED	E51V								
	K48N	M60K								
	D49S	V115I								
	G216A	S201T								
	I218V	E203D								
	L296V	V210G								
	A297G	G211A								
	L299fs	I295L								
	Q300H	A296G								
	R308fs	K299L								
	G309N	A302G								
	D320E	V305I								
	K338I	V311E								
	L361fs	N315K								
		F317V								
		E318D								
		I336K								
OM1433	A18T	G48D				H44Q	**G103S**		**T38S**	A25V
	V24I	E51V					**K62R**		**G74R**	T466A
	L32V	M60K								
	V38A	T99K								
	D39V	Y112F								
	D46N	V115A								
	D46_N47delinsED	F118I								
	K48_D49delinsNS	R185_D186insA								

ID	Porin (influx) regulators			AcrAB-TolC efflux pump regulators						
	**OmpC**	**OmpF**	**OmpR**	**AcrR**	**MarA**	**MarB**	**MarR**	**SoxS**	**SoxR**	**EnvZ**
	V50K	D186G								
	E54K	T187I								
	S85N	A188P								
	N88S	R189E								
	E89D	Y204F								
	N91S	N220D								
	V96L	L221A								
	V104A	Q225E								
	V106A	P226F								
	F109I	L227R								
	G137D	N229fs								
	F149V									
	T155S									
	D156N									
	N165D									
	Q171L									
	T206S									
	D208F									
	Y209F									
	G215V									
	G216A									
	I218V									
	A226D									
	T229N									
	A230T									
	A231fs									
	N235T									
	L296M									
	K317N									
	V319F									
	D320E									
	Q346R									
OM1576	V15A	G48D		S68fs		S5L	**Y137H**			
	N20H	E51V				T24P	**G103S**			
	N47D	M60K				A33G				
	K48N	V115I				V38A				
	D49S	I295L				H44Q				
	V186I	A296G								
	D192G	T298V								
	A226D	F317I								
	T229S	E318D								
	A230fs									
	A284V									
	L296V									
	A297G									
	L299V									
	A297G									
	L299V									
	Q300Y									
	R308fs									
	Q346R									
	N357D									
OM1609		T99K								
		Y204F								
OM1626	V24I	T19A		**V29fs**					**A111T**	
	K48_D49delinsNS	V33L								
	V50K	A39V								
	G137D	V40D								
	N165D	S201T								
		E203N								
		V210G								
		G211A								
		I295L								
		N315K								
		F317A								
		E318_V319delinsDM								
		I336K								

ID	Porin (influx) regulators			AcrAB-TolC efflux pump regulators						
	**OmpC**	**OmpF**	**OmpR**	**AcrR**	**MarA**	**MarB**	**MarR**	**SoxS**	**SoxR**	**EnvZ**
OM1692	V24I	S164Y			S127N	S5L				
	L32V	E184D				A10T				
	V38A	A224fs				A33G				
	D39V	Q225L				H44Q				
	N47D	G228fs								
	D49S	N229K								
	V67I	D278N								
	T155S	V279fs								
	D156N	T298P								
	A277T	K299Q								
	A284fs	A302G								
	L296V									
	A297G									
	N305D									
	R308fs									
	V319									
	K338I									
OM5639	G137D									

fs, mutation leads to frameshift; *Stop codon

Mutations published previously presented with bold font.
