# Supplementary material for: Clinical and molecular characteristics of carbapenem non-susceptible Escherichia coli: A nationwide survey from Oman
Source: PLoS One. 2020 Oct 9;15(10):e0239924. doi: 10.1371/journal.pone.0239924 (PMC7546912; doi:10.1371/journal.pone.0239924)
Supplement: S4 Table — (PDF) [file pone.0239924.s006.pdf]

S4 Table: High-Risk subclones identified in the studied strains.

Clones	Strain	Phylogroup	MER (mm)	CIP (mm)	ParE	ParC	gyrA	PMQR*	β-lactamase enzymes	Virulence genes	Plasmids replicons
**ST131-H30Rx/C2**	OM260	B2	32	6	M74I<br>V76T<br>D77G<br>P85S<br>V87A<br>L89V<br>L91H<br>C92D<br>L94fs<br>V136I<br>I529L	**S80I**<br>**E84V**<br>A192V<br>A471G<br>D475E<br>Q481H	**S83L**<br>**D87N**<br>A828S	*aac* (6')lb-cr<br>*qnr* B4	CTXM15<br>DHA1<br>OXA1	*iha*<br>*pap*<br>yfcV<br>*cnf* 1<br>*cia*<br>*sen* B<br>*sat*<br>*sit* A<br>*chu* A<br>*iuc* C<br>*iut* A<br>*irp2*<br>*fyu* A<br>*kps* E<br>*kps* MII<br>*gad*<br>*usp*<br>*iss*<br>*ter* C<br>*hra*<br>*omp* T	IncF36:A4:B1<br>IncI1 (D)<br>IncN (5)
**ST1193-H64RxC**	OM1136	B2	19	6	L254Q<br>**L416F**	**S80I**	**S83L**<br>**D87N**<br>D678E<br>A828S	*aac* (6')lb-cr	NDM1<br>CTXM15<br>CMY6<br>DHA1<br>OXA1<br>TEM1B	*iha*<br>*pap*<br>*yfc* V<br>*sen* B<br>*sat*<br>*vat*<br>*sit* A<br>*chu* A<br>*iuc* C<br>*iut* A<br>*irp* 2<br>*fyu* A<br>*kps* E<br>*kps* MII<br>*neu* C<br>*gad*<br>*usp*<br>*ter* C<br>*omp* T	IncF-: A1:B10<br>IncA/C2<br>IncQ1<br>ColBS512<br>Col156
**ST410-H24RxC**	OM561	C	15	6	none	none	**S83L**<br>**D87N**	*aac* (6')lb-cr	NDM7<br>CTXM15<br>OXA1	*Ipf* A<br>*sen* B<br>*sit* A<br>*iuc* C<br>*iutA*<br>*irp* 2<br>*fyu* A<br>*gad*<br>*ter* C	IncF31:A4:B1<br>IncX3<br>IncL/M<br>ColpVC
	OM1273	C	22	6	L254Q<br>**L416F**<br>I529L<br>S458A	**S80I**	**S83L**<br>**D87N**	*aac* (6')lb-cr<br>*qnr* S1	OXA181<br>CTXM15<br>CMY2<br>OXA1<br>TEM1B	*Ipf* A<br>*gad*<br>*ter* C	IncF1:A1:B49<br>IncX3<br>IncQ1<br>ColKP3
	OM1301	C	8	6	none	**S80I**	none	*aac* (6')lb-cr	NDM5<br>OXA181<br>CTXM15<br>CMY2<br>OXA1<br>TEM1B	*Ipf* A<br>*gad*<br>*ter* C	IncF1:A1:B49<br>IncX3<br>IncY<br>IncQ1<br>ColBS512<br>ColKP3

*PMQR, plasmid-mediated quinolone resistance; MER, meropenem; CIP, ciprofloxacin.
Chromosomal mutations known to cause fluoroquinolones resistance were marked with bold font.
